# Trends in dental caries of deciduous teeth in Iran: a systematic analysis of the national and sub-national data from 1990 to 2017

**DOI:** 10.1186/s12903-022-02634-z

**Published:** 2022-12-23

**Authors:** Shervan Shoaee, Sahar Saeedi Moghaddam, Masoud Masinaei, Ahmad Sofi-Mahmudi, Hossein Hessari, Mohammad-Hossein Heydari, Erfan Shamsoddin, Mahboubeh Parsaeian, Anooshe Ghasemian, Bagher Larijani, Hossein Fakhrzadeh, Farshad Farzadfar

**Affiliations:** 1 Elderly Health Research Center, Endocrinology and Metabolism Population Sciences Institute, Tehran University of Medical Sciences, Tehran, Iran; 2 Non-Communicable Diseases Research Center, Endocrinology and Metabolism Population Sciences Institute, Tehran University of Medical Sciences, 10, Al-e-Ahmad and Chamran Highway Intersection, Tehran, 1411713136 Iran; 3 Kerman Oral and Dental Diseases Research Center, Kerman University of Medical Sciences, Kerman, Iran; 4 Cochrane Iran Associate Centre, National Institute for Medical Research Development (NIMAD), Tehran, Iran; 5 Research Center for Caries Prevention, Dentistry Research Institute, Tehran University of Medical Sciences, Tehran, Iran; 6 School of Dentistry, Shahid Beheshti University of Medical Sciences, Tehran, Iran; 7 Department of Epidemiology and Biostatistics, School of Public Health, Tehran University of Medical Sciences, Tehran, Iran; 8 Endocrinology and Metabolism Research Center, Endocrinology and Metabolism Clinical Sciences Institute, Tehran University of Medical Sciences, 10, Al-e-Ahmad and Chamran Highway Intersection, Tehran, 1411713111 Iran

**Keywords:** Dental caries, Iran, Tooth decay, Spatio-temporal analysis, Tooth loss

## Abstract

**Background:**

Dental caries is the most prevalent child affliction in the world and can be reduced through effective preventive interventions. To plan cost-effective interventions, clear and integrated data are needed. This study has been designed to overcome the lack of national trend in deciduous dental caries in Iran.

**Objective:**

To estimate the dental caries trend in deciduous teeth in the Iranian population at different ages from 1990 to 2017.

**Methods:**

From 1990 to 2017 a literature search about dmf and its components (decayed, missed, and filled tooth, abbreviated as dt, mt, and ft) as well as dental caries was done in the Iranian population in three English (PubMed, Web of Science, and Scopus) and three national databases (in Persian). All eligible national oral health surveys in these 28 years were included. National dmft data were categorised based on age (1–4, 5–9, and 10–14), sex, province and year. The final trends were estimated using an age-spatio-temporal hierarchical model. We used the bootstrap method in multilevel models to predict the uncertainty interval (UI) of the modelled results. Finally, the estimations of dmft, dt, mt, and ft with a 95% UI were reported from 1990 to 2017.

**Results:**

Almost 22% of the Iranian deciduous teeth were involved with dental caries in 1990 [dmft = 4.37; (95% UI 2.23, 6.62)] which more than 83% of it was dt [3.64 (1.53, 5.88)] and less than 7% was ft [0.30 (0.06, 0.65)]. During 1990–2017, dmft increased by more than 15% [in 2017, dmft = 5.03 (2.82, 7.29)]. The highest increase was seen in dt which was more than 17% [in 2017, dt = 4.27 (1.96, 6.57)].

**Conclusion:**

Increasing dental caries among Iranian children over 28 years shows that oral health policies in Iran need critical evaluation. We need cost-effective nationwide interventions (e.g., supervised tooth brushing and improving dietary habits) and training well-experienced intermediate manpower (e.g., dental hygienists) to reduce dental caries.

**Supplementary Information:**

The online version contains supplementary material available at 10.1186/s12903-022-02634-z.

## Background

Despite being largely preventable and due to its ubiquitous nature, child dental caries remains the most prevalent child affliction around the globe, affecting up to 60–90% of school-aged children in most industrialised countries [1–4]. This results in a high economic and disease burden on countries. Iran is no exception: child dental caries has been estimated to have caused more than 1500 years lived with disability in 2017 [4] and more than $10 M in productivity losses in 2015 [5]. With this considerable burden, oral disorders tend to be overlooked among other health conditions and are rarely seen as a priority in health policy [1, 6, 7]. Other physiologically influential factors include factors affecting tooth germ formation, natural protective factors, such as saliva, and exposure to a low fluoride level [8–10].

Oral diseases are chronic and progressive in nature [6, 11]. For example, dental caries affects very young children but is a lifelong condition that tracks across adolescence, adulthood, and into later life [6]. Poor oral health can negatively impact a child’s ability to eat, speak, sleep, and socialise, resulting in adverse impacts later in life [12, 13]. In addition to affecting children’s quality of life [14] and school performance [15], dental caries places a considerable burden on health care provision. For instance, most hospital admissions for children in England between 1997 and 2006 were primarily due to dental caries [16].

After 1960, an evident decline in caries prevalence occurred in Western European countries [17]; children’s and adolescents’ habit of brushing their teeth with fluoride toothpaste was the most crucial factor in this decline [18, 19]. However, this is not the case in developing countries where the oral health system is not fully established.

In developed countries, regular child dental health surveys have been established several decades ago (such as in the UK [20], Australia [21], and Canada [22], but in developing countries there are no regular, precise, and adequate national data in the literature. For example, the three latest child dental health surveys in Iran date back to 2004, 2013, and 2016, and the results of the survey in 2016 showed that the mean decayed, missing, and filled teeth (dmft) of Iranian school children aged 6 and 12 was 5.84 which is seven and nearly two times that of the UK’s [23] and Turkey’s [24] children, respectively. This highlights the need to make or change policies to reduce dental caries in children.

However, policy-making could not be done effectively without proper data and understanding of the current situation, which helps us in making informed decisions and improving future policy planning. This study aims at estimating the national and sub-national dental caries trend in deciduous teeth among the Iranian population at different ages by sex from 1990 to 2017. The findings of this descriptive report will serve as a baseline for monitoring the progress of policies seeking to address oral health status in Iran.

## Methods

The Burden of Oral Disorders (BOD) is a part of a more extensive study (National And Sub-national Burden of Diseases: NASBOD) aiming to assess dental caries, periodontal disease, and severe tooth loss. We scrutinised all the relevant literature; either published, unpublished, or grey literature. After that, we ascertained the eligible studies using three phases including title, abstract, and full-text. Data extraction was performed after assessing the quality of data using a form based on the STrengthening the Reporting of OBservational studies in Epidemiology (STROBE) Statement [25]. Finally, data analyses and estimations were conducted using an age-spatio-temporal hierarchical model. The protocol for this study has been published earlier [26]. Full details of the methods of the current study are available in the protocol. Here, we will discuss the most critical aspects of our methodology more rigorously.

### Disease selection and definitions

Based on the Global burden of diseases, injuries, and risk factors (GBD) and NASBOD studies, and through expert panel meetings, dental caries, periodontal diseases, and severe tooth loss were selected for the current study. The definition of dental caries was adopted from definitions given by World Health Organisation (WHO) (2013) [27], Medical Subject Headings (MeSH, www.ncbi.nlm.nih.gov/mesh), and International Classification of Diseases, 10th Revision (ICD10, www.who.int/classifications/icd/en), as follows: diagnosis of caries in the D3 area; dentinal exposure; enamel softening; unsupported enamel; and, discolouration in the inter-dental area.

### Data sources

Several data sources were used in the study that could be categorised into two main groups:

A. Published, unpublished, and grey literature; and
B. National data.

#### A. Published, unpublished and grey literature

Literature published in English and Persian were searched in these databases: PubMed, ISI (Web of Science), Scopus as international databases, and IranMedex, SID (Scientific Information Database), and IranDoc as domestic databases. To find unpublished data, key informants and authors were contacted. Theses, reports of research projects, and governmental reports were also evaluated and included in the study.

The literature review was done based on a combination of the following search terms: prevalence, incidence, general mortality, cause-specific mortality, remission rate, age of onset and disability weights, with names of the six diseases. Full details of the search terms are available in Additional file 1: Appendix 1, page 4.

##### Selection criteria

*Type of study* All the cross-sectional studies and results of the baseline survey of cohort studies were included. Moreover, all national, provincial, district and community studies reported the prevalence of dental caries or dmft mean were included in the study. Case reports, case series, clinical trials with small sample sizes, and non-population-based surveys were excluded.

*Study population* The population was representative of Iranian children population with deciduous teeth. Populations with special health conditions who are not healthy, specific population groups based on their (un)employment, occupation or education status, and also immigrants were excluded.

*Sampling method* Studies that did not report the sampling method or did not report the sampling method clearly were excluded.

##### Selection of searched articles

After the searching phase, found articles were exported to the reference management software (EndNote X7.0; Thomson Reuters, Toronto, Ontario, Canada). Final articles were selected according to the inclusion and exclusion criteria in three phases. In case of disagreement, reviewers discuss the doubtful articles to reach an agreement and select them.

*Title phase* Two reviewers scanned all titles according to the inclusion criteria. Titles in doubt were also included.

*Abstract phase* The same two reviewers read all abstracts according to the inclusion criteria together. Articles with unclear methods were also included.

*Full-text phase* To finalise the selection phase, the reviewers evaluated all remaining articles to determine if they are eligible.

Full texts were obtained via referring to the Tehran University of Medical Sciences’ digital library, contacting corresponding authors, or accessing the article published.

##### Quality assessment and data extraction

To assess the quality of articles with different sampling and measuring methods, based on the criteria of the study characteristic sheet, a new comprehensive quality assessment form with defined ranking scores for the systematic review, was used through four steps.

1. First, a data extraction form was developed based on the study characteristics sheet with defined criteria such as study ID, citation, corresponding author’s characteristics, study year, study sources, study design, the scope of the study, level of study, sample weight, sampling quality and measurement quality (Additional file 1: Appendix 2, page 5). The quality assessment form included the following criteria: ID, disease group, the study scope, study level, study type, power of the study, sampling method, sex, province, location, sample size, sample weight, practical disease definition, disease exploration, validity, intra-observer reliability, inter-observer reliability, examiner level and the number of examiners (Additional file 1: Appendix 3, page 7).
2. Next, each criterion was defined and explained precisely. The form was designed in Epi-Info 7 in Microsoft Access 2013 format, and ranking scores were given to the articles to assess their qualities.
3. To validate the form’s face and content, the expert panel and focus group discussion method were used, and the form was finalised.

Finally, the data extraction sheet was used to summarise study data based on sex, age, and sample size in related age and sex groups, prevalence, incidence, mean, standard deviation, standard error, and confidence intervals (Additional file 1: Appendix 2 B, page 6).

#### B. National data

*National oral health surveys* These surveys were designed to collect national and provincial data about oral health and its risk factors in Iran through oral health examinations. Data from national oral health surveys in 1998, 2002, 2004, 2013, and 2016 were included.

*National health surveys* These surveys are repeated multipurpose national surveys covering all Iranian households. These repeated surveys were implemented to evaluate indicators of health and illness. In 1990 and 1999, the survey included oral health data for different age groups of the Iranian population.

##### Statistical methods and analysis

The methods used in this study have been used for estimation of the burden of diseases in Iran previously [28, 29]. After cleaning the relevant data, dmft and its components (dt, mt, and ft) were categorised based on age, sex, year, and province and then aggregated. We chose 5-year-based groups (except for the first age-group, 1–4, which contains children from 4 years) to have comparable data with the GBD study [30]. Another reason was that as we used NASBOD study covariates, we should have had similar age-groups as that study which was 5-year-based.

We addressed the misalignment problem due to changes in the changes in administrative divisions between provinces and districts, and new introduced provinces and districts.

For analysing the categorised and cleaned data, firstly, a model was designed for dmft and each of its components using a random intercept mixed-effects model [31] with these independent variables: mean years of schooling (derived from Iran’s household expenditure and income survey), wealth index (derived from Iran’s household expenditure and income survey), and mean weight of each age within each age group (derived from Iran’s regular national census). Next, using the age-spatio-temporal model, temporal and spatial correlations, as well as correlations between age groups, were addressed.

Weighting components were defined as follows:

- Spatial component ($W_{{L_{ij} }}$): if two provinces were adjacent, the weight was considered to be 1; otherwise, 0 was considered as the spatial weight;
- Temporal component ($W_{{T_{ij} }}$): similar to the LOESS (locally estimated scatterplot smoothing) regression, we used the cubic power: $W_{{T_{ij} }} = { }\left( {1 - \left( {\frac{{\left| {i - j} \right|}}{{ArgMax\left( {\left| {i - j} \right| + 1} \right)}}} \right)^{\lambda } } \right)^{3}$.
  o $\lambda$ is a smoothing parameter. Based on the previous studies, this parameter was considered to be 2.
- Age component ($W_{{A_{ij} }}$): assuming that increased difference between two age groups is associated with a reduction in their weight, defined as $W_{{A_{ij} }} = { }\frac{1}{{e^{{\varpi { }\left| {i - j} \right|}} }}$ in the matrix.
  o $\varpi$ is a smoothing parameter such that with lower values of it, smoothing will be higher. Based on the previous studies, this parameter was considered to be 1.

To predict the uncertainty interval (UI) of the modelled results, we used a bootstrap method for multilevel models [32, 33]. Using this method, first, we extracted the distribution of sampling fixed (such as sex, mean years of schooling, wealth index, and mean weighting of the age group) and random (province and year) effects and then fitted values of the model were estimated. The advantage of using this method over other bootstrapping methods is that it considers all the uncertainties (in fixed or random effects) in the model. Furthermore, it is more rapid [34, 35].

To calculate the age-standardised dmft and its components at the province level, each age group was standardised based on the report of the population and housing census conducted by Iran’s Statistical Center, in 2016 [36].

We reported estimations of dmft and its components from 1990 to 2017 with 95% UI. Statistical analyses were performed using STATA v14 and R v3.5.2 (R Core Team, R Foundation for Statistical Computing, Vienna, Austria; http://www.R-project.com) with Age-Spatial–Temporal Model v0.1.0 (AST) package (https://cran.r-project.org/web/packages/AST/index.html).

Our article complies with the Guidelines for Accurate and Transparent Health Estimates Reporting (GATHER). Our full GATHER checklist is available in the Additional file 1: Appendix 5.

## Results

### Literature review

**Fig. 1:**
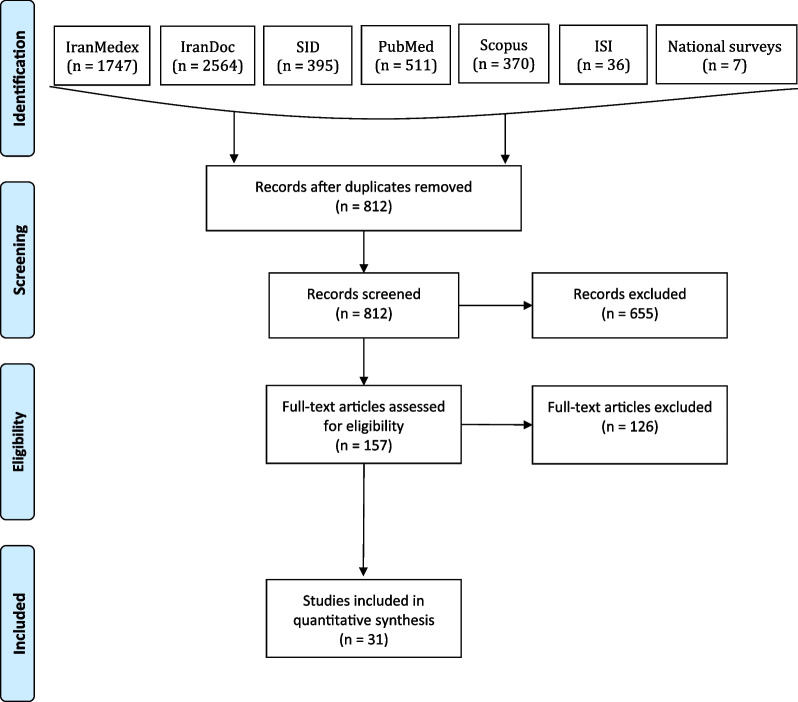
The flow diagram of systematic search

At first, we reached 5983 articles (1930 English and 4053 Persian) reporting dental caries among all ages. Searching in other sources (e.g., grey literature) provided us with six additional records. By removing duplicates, 812 articles remained. After screening based on the inclusion and exclusion criteria, 31 studies that had dmft data were included (Fig. 1).

### National dmft in all ages

On average, Iranian children had caries experience in almost 22% of their deciduous teeth in 1990 [dmft = 4.37; (95% UI 2.23, 6.62)] which more than 83% of it was decayed teeth [dt = 3.64 (1.53, 5.88)] and less than 7% was filled teeth [ft = 0.30 (0.06, 0.65)]. During 1990–2017, dmft increased by more than 15% (in 2017, dmft = 5.03 [(2.82, 7.29)]. The highest increase was seen in dt which was more than 17% [in 2017, dt = 4.27 (1.96, 6.57)]. The number of missing teeth decreased slightly [1990 mt = 0.44 (0.17, 0.84)], 2017 mt = 0.43 [(0.15, 0.82)]. More information is illustrated in Table 1.

**Table 1:** Mean number of decayed, missing, and filled teeth and caries experience (dmft) by age group for Iran, 1990–2017, by sex

Age group	Year	dmft			dt			mt			ft		
		Both	Female	Male	Both	Female	Male	Both	Female	Male	Both	Female	Male
1–4	1990	2.22 (0.27, 4.44)	1.99 (0.13, 4.2)	2.45 (0.41, 4.67)	1.98 (0.09, 4.38)	1.76 (0.01, 4.16)	2.19 (0.16, 4.59)	0.01 (0, 0.36)	0 (0, 0.32)	0.02 (0, 0.4)	0.23 (0.03, 0.61)	0.23 (0.03, 0.61)	0.23 (0.04, 0.61)
	2000	2.65 (0.52, 4.94)	2.45 (0.35, 4.74)	2.84 (0.67, 5.13)	2.36 (0.08, 5.37)	2.16 (0.01, 5.25)	2.54 (0.15, 5.49)	0 (0, 0.37)	0 (0, 0.34)	0.01 (0, 0.4)	0.29 (0.05, 0.77)	0.29 (0.05, 0.78)	0.29 (0.05, 0.76)
	2010	2.88 (0.71, 5.18)	2.74 (0.58, 5.04)	3.01 (0.84, 5.32)	2.56 (0.18, 5.36)	2.43 (0.1, 5.27)	2.7 (0.26, 5.45)	0 (0, 0.34)	0 (0, 0.32)	0 (0, 0.36)	0.31 (0.07, 0.76)	0.32 (0.07, 0.77)	0.31 (0.07, 0.75)
	2017	3.01 (0.92, 5.18)	2.91 (0.82, 5.08)	3.1 (1.01, 5.27)	2.7 (0.4, 5.11)	2.61 (0.32, 5.03)	2.79 (0.47, 5.19)	0 (0, 0.31)	0 (0, 0.3)	0 (0, 0.32)	0.3 (0.07, 0.69)	0.3 (0.07, 0.69)	0.3 (0.07, 0.68)
5–9	1990	4.34 (2.17, 6.57)	4.13 (1.96, 6.34)	4.55 (2.37, 6.78)	3.64 (1.45, 5.85)	3.48 (1.27, 5.7)	3.8 (1.62, 5.99)	0.38 (0.03, 0.8)	0.33 (0, 0.76)	0.42 (0.05, 0.85)	0.32 (0.08, 0.67)	0.33 (0.07, 0.67)	0.32 (0.08, 0.67)
	2000	4.78 (2.55, 7.06)	4.62 (2.4, 6.9)	4.92 (2.7, 7.21)	4.05 (1.62, 6.47)	3.92 (1.48, 6.37)	4.16 (1.76, 6.57)	0.38 (0.02, 0.85)	0.34 (0, 0.82)	0.41 (0.03, 0.88)	0.35 (0.09, 0.74)	0.36 (0.09, 0.74)	0.35 (0.1, 0.73)
	2010	4.97 (2.78, 7.24)	4.89 (2.7, 7.15)	5.05 (2.85, 7.32)	4.22 (1.88, 6.55)	4.16 (1.81, 6.49)	4.29 (1.94, 6.61)	0.38 (0.02, 0.83)	0.36 (0, 0.81)	0.4 (0.03, 0.85)	0.37 (0.11, 0.74)	0.37 (0.1, 0.74)	0.36 (0.11, 0.73)
	2017	5.06 (2.99, 7.2)	5.04 (2.97, 7.17)	5.08 (3.01, 7.22)	4.34 (2.17, 6.48)	4.32 (2.15, 6.46)	4.36 (2.19, 6.49)	0.37 (0.02, 0.79)	0.36 (0, 0.78)	0.38 (0.03, 0.8)	0.35 (0.11, 0.7)	0.36 (0.11, 0.7)	0.35 (0.11, 0.69)
10–14	1990	6.42 (4.15, 8.75)	6.23 (3.98, 8.54)	6.6 (4.32, 8.94)	5.2 (3.01, 7.33)	5.05 (2.87, 7.17)	5.33 (3.13, 7.48)	0.9 (0.48, 1.32)	0.86 (0.4, 1.27)	0.95 (0.52, 1.37)	0.32 (0.06, 0.67)	0.33 (0.06, 0.67)	0.32 (0.07, 0.66)
	2000	6.83 (4.41, 9.3)	6.73 (4.3, 9.18)	6.93 (4.51, 9.41)	5.55 (3.11, 7.92)	5.46 (3.01, 7.84)	5.63 (3.21, 7.98)	0.95 (0.48, 1.42)	0.93 (0.5, 1.4)	0.98 (0.51, 1.45)	0.33 (0.06, 0.71)	0.34 (0.06, 0.72)	0.32 (0.07, 0.7)
	2010	7.01 (4.5, 9.56)	7 (4.48, 9.56)	7.02 (4.52, 9.56)	5.71 (3.2, 8.14)	5.7 (3.16, 8.16)	5.72 (3.25, 8.12)	0.96 (0.47, 1.44)	0.95 (0.5, 1.43)	0.96 (0.49, 1.44)	0.34 (0.07, 0.73)	0.35 (0.07, 0.75)	0.33 (0.07, 0.72)
	2017	7.06 (4.57, 9.56)	7.12 (4.63, 9.65)	6.99 (4.51, 9.48)	5.79 (3.32, 8.18)	5.84 (3.31, 8.28)	5.75 (3.32, 8.09)	0.92 (0.45, 1.39)	0.93 (0.4, 1.4)	0.92 (0.45, 1.38)	0.34 (0.07, 0.73)	0.35 (0.08, 0.75)	0.33 (0.07, 0.71)
All ages	1990	4.37 (2.23, 6.62)	4.15 (2.04, 6.39)	4.58 (2.41, 6.84)	3.64 (1.53, 5.88)	3.45 (1.39, 5.7)	3.81 (1.67, 6.05)	0.44 (0.17, 0.84)	0.4 (0.1, 0.79)	0.47 (0.19, 0.88)	0.3 (0.06, 0.65)	0.3 (0.05, 0.65)	0.29 (0.07, 0.65)
	2000	5.09 (2.81, 7.45)	4.95 (2.68, 7.31)	5.23 (2.94, 7.59)	4.25 (1.85, 6.79)	4.12 (1.75, 6.7)	4.36 (1.95, 6.88)	0.52 (0.2, 0.97)	0.5 (0.2, 0.94)	0.54 (0.22, 0.99)	0.33 (0.07, 0.73)	0.33 (0.07, 0.75)	0.32 (0.07, 0.72)
	2010	5.08 (2.78, 7.46)	5.01 (2.71, 7.39)	5.15 (2.85, 7.52)	4.26 (1.85, 6.77)	4.2 (1.79, 6.73)	4.33 (1.91, 6.8)	0.47 (0.18, 0.9)	0.46 (0.2, 0.89)	0.48 (0.18, 0.92)	0.34 (0.08, 0.74)	0.35 (0.08, 0.75)	0.34 (0.08, 0.73)
	2017	5.03 (2.82, 7.29)	5.01 (2.8, 7.28)	5.04 (2.83, 7.3)	4.27 (1.96, 6.57)	4.25 (1.93, 6.57)	4.28 (1.99, 6.57)	0.43 (0.15, 0.82)	0.42 (0.1, 0.82)	0.43 (0.15, 0.83)	0.33 (0.09, 0.7)	0.34 (0.09, 0.71)	0.33 (0.08, 0.69)

### 1–4 age group

**Fig. 2:**
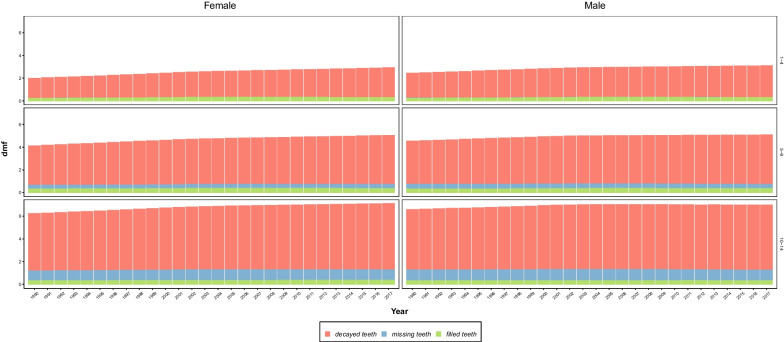
Stacked bar chart of decayed, missing, and filled teeth from 1990 to 2017

From 1990 to 2017, dmft has increased continuously for 1–4 age group [from 2.22 (0.27, 4.44) to 3.01 (0.92, 5.18)], respectively. The number of dt has been responsible for the major part of dmft and increased from 1.98 (0.09, 4.38) in 1990 to 2.70 (0.40, 5.11) in 2017. However, the number of mt has decreased slightly in this period). The number of ft has increased [(0.23 (0.03, 0.61) in 1990 vs. 0.30 (0.07, 0.69) in 2017]; however, the highest number of ft has been estimated to be 0.32 in 2004–2009 (Table 1). Boys had higher dmft than girls in the whole period [2.45 (0.41, 4.67) vs. 1.99 (0.13, 4.20) in 1990; 3.10 (1.01, 5.27) vs. 2.91 (0.82, 5.08) in 2017, respectively]. Boys also had a higher number of dt [2.19 (0.16, 4.59) vs. 1.76 (0.01, 4.16) in 1990; 2.79 (0.47, 5.19) vs. 2.61 (0.32, 5.03) in 2017] (Fig. 2 and Table 1).

### 5–9 age group

Our analysis showed that dmft has also increased continuously in this age group from 1990 to 2017 [4.34 (2.17, 6.57) and 5.06 (2.99, 7.20), respectively]. Like the 1–4 age group, the number of dt was the main component of dmft with 3.64 (1.45, 5.85) in 1990 and 4.34 (2.17, 6.48) in 2017, which increased continuously. The number of mt was almost fixed during 1990–2017 [0.38 (0.03, 0.80) vs. 0.37 (0.02, 0.79)] whereas the number of ft has been continuously increasing from 1990 to 2006 [0.32 (0.08, 0.67) vs. 0.37 (0.11, 0.76)] and then continuously decreasing since then to 0.35 (0.11, 0.70) in 2017 (Table 1). Boys had higher dmft [4.55 (2.37, 6.78) vs. 4.13 (1.96, 6.34) in 1990; 5.08 (3.01, 7.22) vs. 5.04 (2.97, 7.17) in 2017], number of dt [3.80 (1.62, 5.99) vs. 3.48 (1.27, 5.70) in 1990; 4.36 (2.19, 6.49) vs. 4.32 (2.15, 6.46) in 2017], and number of mt [0.42 (0.05, 0.85) vs. 0.33 (0.01, 0.76) in 1990; 0.38 (0.03, 0.80) vs. 0.36 (0.02, 0.78) in 2017]. However, the number of ft was slightly higher in girls [0.32 (0.08, 0.67) vs. 0.33 (0.07, 0.67) in 1990; 0.35 (0.11, 0.69) vs. 0.36 (0.11, 0.70) in 2017] (Fig. 2 and Table 1). The dmft trend and further information regarding deciduous teeth are summarized in Fig. 1 and Table 1, respectively. 

### 10–14 age group

The highest dmft in these three age groups was seen in 10–14 age group with dmft = 6.42 (4.15, 8.75) in 1990 and dmft = 7.06 (4.57, 9.56) in 2017. Similar to the other two age groups, the major part of dmft was attributable to dt [5.20 (3.01, 7.33) in 1990; 5.79 (3.32, 8.18) in 2017[. The number of mt increased till 2005 [0.90 (0.48, 1.32) in 1990; 0.97 (0.49, 1.45) in 2005] and then decreased to 0.92 (0.45, 1.39) in 2017. The number of ft was slightly increased; from 0.32 (0.06, 0.67) in 1990 to 0.34 (0.07, 0.73) in 2017 (Table 1). While dmft was higher in boys in 1990 [6.60 (4.32, 8.94) vs. 6.23 (3.98, 8.54)], dmft in girls had a higher rise than in boys, and in 2017 girls had higher dmft [6.99 (4.51, 9.48) vs. 7.12 (4.63, 9.65)]. Whereas the number of dt was higher in boys in 1990 [5.33 (3.13, 7.48) vs. 5.05 (2.87, 7.17)], girls experienced a higher pace of increase, resulting in a higher number of dt than boys in 2017 [5.75 (3.32, 8.09) vs. 5.84 (3.31, 8.28)]. In 1990, the number of mt was higher for boys [0.95 (0.52, 1.37) vs. 0.86 (0.44, 1.27)] and increased till 2003 for boys [0.99 (0.51, 1.46)] and then decreased to 0.92 (0.45, 1.38) in 2017 whereas there was a continuous increase for girls [0.93 (0.44, 1.40) in 2017]. The number of ft was slightly higher for girls in 1990 [0.32 (0.07, 0.66) vs. 0.33 (0.06, 0.67)], and both sexes experienced increasing trend till 2017 [0.33 (0.07, 0.71) for boys, 0.35 (0.08, 0.75) for girls] (Fig. 2 and Table 1).

### Sub-national dmft

**Fig. 3:**
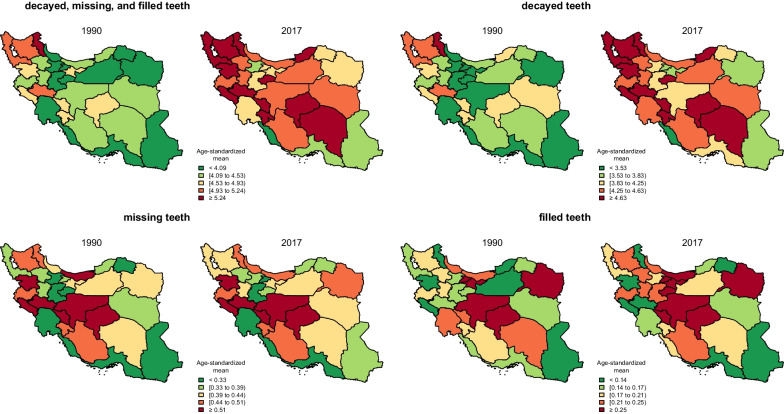
Map chart for dmft and its components in 1990 and 2017 (age-standardised)

**Fig. 4:**
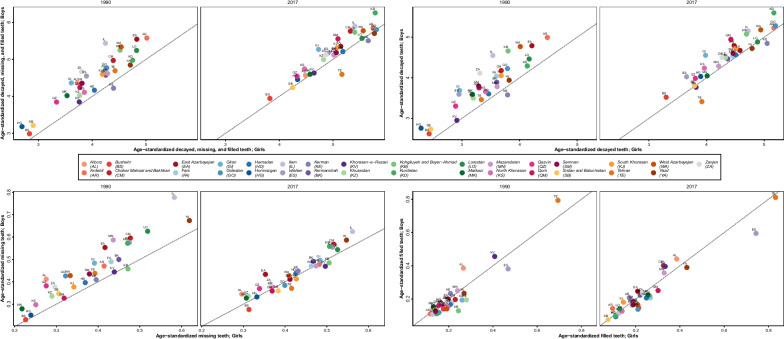
Comparison of sub-national age-standardized dmft in 1990 and 2017 by sex. *The dotted line indicates the cut off for gender disparity. Those provinces above the line had higher dmft, dt, mt, and ft among boys in 1990 and 2017

All provinces experienced an increase in age-standardised dmft 1990–2017. Southern provinces showed the least age-standardised dmft [lowest in Bushehr with dmft in 1990: 2.91 (1.13, 5.16), and in 2017: 3.89 (1.73, 6.17)] whereas north-western and western provinces had the highest mean dmft [the highest is estimated in Ardabil with 5.30 (3.14, 7.53) in 1990 and in Kurdistan with dmft 6.03 (3.78, 8.29) in 2017]. When examining dmft components, the mean number of dt has increased in every province while the mean number of mt and ft has decreased in seven and nine provinces, respectively. While the dmft has been increased during 1990–2017, the divergence between provinces has decreased in dmft, dt, and mt. For instance, in 1990, the difference between the highest and lowest dmft was 2.39 (between Ardabil and Bushehr), whereas, in 2017, this difference decreased to 2.14 (between Kurdistan and Bushehr). However, the sub-national divergence of ft has increased, from 0.63 in 1990 (between Tehran and Ardabil) to 0.75 in 2017 (between Tehran and Sistan and Baluchistan). More information about sub-national dmft, gender disparity, and its components is shown in Figs. 3, 4 and Additional file 1: Appendix 4.

## Discussion

This study provides a unique opportunity to look at children’s oral health and disease in Iran over three decades. This paper has focused on one disease, caries. Dental caries is the most prevalent health condition globally, imposing a high health and economic burden on countries. Knowing the past situation and comparing it with the current status is crucial to evaluating nationwide oral health programs (if any) and providing a better point-of-view for further oral health policies. However, there are no publicly available nationwide trend data regarding child dental health in Iran, and this study endeavoured to fill in this gap.

The results highlight a high mean number of dmft across all child age groups, which has continuously increased since 1990 (more than 15%). The number of mt has been almost unchanged during these three decades, and an increased number of dt and ft is responsible for increasing dmft. The highest increase in dmft and dt was in 1–4-years age group with more than 35% and 36% increase, respectively. Overall, the highest dmft was found among the 10–14-years age group which was not unexpected, as dmft is a cumulative index.

As shown in Fig. 3, this increase in dmft gradually spreads from northwest to middle, south, and southeast of Iran and can be attributed to the shift in dietary habits of the Iranian children. This shift could also be worsened by the low frequency of daily tooth brushing and flossing among children in Iran [37, 38]. According to one study, only 2.7% of children (6 to 14 years old) brush and floss on a daily basis [39]. As depicted in Fig. 4, dmft was higher among boys compared to girls in 1990 in almost all provinces. Despite the fact that dmft increased among all age and both sexes, this difference remained until 2017. Also, an interesting finding is that although dt is higher among boys in almost all age groups and all provinces, ft is not significantly different among them. This means that girls experience tooth decay less than boys, and when they do so, they receive better or at least equal treatment. It should be noted that our data did not include any study on children with disabilities, and as this vulnerable population experience higher rate of tooth decay and also have lower access to care [40], future national surveys should include this population accordingly.

While the number of dental schools and dentists has increased dramatically during the last two decades [41], seemingly it had little or no positive impact on Iranian children’s dental health, at least in the short term. In contrast, dmft has increased. The number of ft, which is a result of dental procedures, has increased slightly. The reasons could be various, including families’ better economic situation, a shift in families’ mindset towards the importance of their children’s dental health, and a higher number of available dentists [41]. However, the mean of dt is more than 13 times the ft mean in 2017, which implies the oral health system has failed to provide adequate oral health care to Iranian children.

National and international studies have shown that increasing child dental caries burden has not occurred in every country. For example, the GBD 2017 study has stated that the Global age-standardized prevalence [− 7.9% (− 6.6%, − 9.8%)], incidence [− 2.2% (− 0.4%, − 3.9%)], and Disability-Adjusted Life Years (DALYs) [− 9.0% (− 7.3%, − 11.0%)] rates of untreated caries in deciduous teeth has decreased from 1990 to 2017 [4]. The UK’s child dental health survey 2013 has also shown that the prevalence of caries and dmft for children in England, Wales and Northern Ireland is continuing to decrease but the rate is slowing [42]; from 4.0 in 1973 to 0.7 in 2013 for 5-year-old children [20]. The mean dmft has also decreased in German 6- to 7-years-olds, from 2.89 in 1994/95 to 1.73 in 2016 [43]. Not only in industrialized countries, but some developing countries have also reported that the mean dmft of their children has decreased. For example, based on a survey in Turkey, it has been reported that the mean dmft for 5-year-olds has decreased from 1988 to 2004 (dmft = 3.7 in 2004) [24] which was much lower than dmft for the 5–9-years age group in 2000 and 2010 in Iran (4.78 and 4.97, respectively). However, some middle-eastern countries have also high dmft; for example, in a meta-analysis, it was estimated that the mean dmft for Saudi Arabian children was 5.0 [44]. In some other middle-eastern and north African countries such as Sudan, Tunisia, and Libya, dmft was lower. Furthermore, unlike Iran, the dmft trend in Iraq and Kuwait has been decreasing [45]. This indicates the unfavorable child dental health situation in Iran, and oral health policy-makers should consider that.

There is strong evidence that fluoride can prevent dental caries in children. Fluoride can be delivered via drinking water [46], toothpaste [18], and mouth rinses [47]. There was a fluoride mouthwash program in Iranian schools during the 2000s but it did not continue and was never evaluated in terms of cost-effectiveness [48]. It is known that supervised use of fluoride toothpaste and mouth rinses will result in a higher decrease in dental caries in children [18, 47]. In-office preventing measurements such as fluoride varnishes and gels and pit-and-fissure sealants also decrease child dental caries in the population [47–49]. Furthermore, oral health education alone or in combination with supervised tooth brushing with fluoridated toothpaste could have a beneficial effect on dmft [50]. Lowering the amount of sugar in the diet is also linked with lower dental caries [51]. Public Health England suggests a targeted supervised tooth brushing program, targeted fluoride varnish program, water fluoridation, targeted provision of toothbrushes and paste by post, and targeted toothbrushes and paste provision by post and by health visitors as programs that can effectively reduce tooth decay in 5-year-olds. Among them, water fluoridation has the highest return on investment; £12.71 return on investment after five years and £21.98 return on investment after ten years for each pound spent. It is not surprising that our study showed that child dental caries is lowest in Bushehr (dmft = 3.89), Sistan and Baluchistan (dmft = 4.24), and Hormozgan (dmft = 4.40) provinces where the amount of fluoride in their drinking water is naturally high [52]. Finally, dental hygienists have a critical role in prevention and health promotion [53, 54] whom are not trained regularly in Iran for more than 10 years with no policy to utilize them in the oral health system.

Although our study is not an analytical one comparing provinces based on socioeconomic factors, Kurdistan with the second-lowest human development index (HDI) had the highest dmft versus Sistan and Baluchistan with the lowest HDI had the second-lowest dmft in Iran [55, 56]. Considering the pattern of sugar consumption and water fluoride has differed among Iranian provinces, perhaps it hides the direct effect of the HDI and can be one of the related causes of this conflict. It has been previously shown that child dental caries follows a socioeconomic gradient [57, 58] and any oral health promotion program considers target socioeconomic factors [59]. These factors are so important that even with providing free dental care services for everyone and equal attendance, there would still be inequalities in dental caries experience [60]. At the same time, they could make inequalities in attendance as well [61]. Hence, we should make a change from providing dental care to a plan with mostly preventive oral health programs.

Dental caries not only affects body weight, growth and quality of life in children [62] but also can affect their later life [63]. Furthermore, dental caries shares common risk factors with various systemic conditions such as obesity, as in both conditions, sugar consumption is the main risk factor [64–66]. Data from Iran’s Agriculture Ministry show that the import of raw sugar was five-fold in 2006 compared to the previous year, and for the consequent years, sugar import has been higher than its export. We believe this could be one of the main causes of increasing dmft of the Iranian children and all other age groups. It is previously suggested to adopt a common risk factor approach in oral health policies [67, 68].

### Strengths

In this paper, the most extensive collections of dental caries of deciduous teeth estimates are provided. By applying a comprehensive systematic search, all qualified published and unpublished sources, in both English and Persian in addition to all national surveys were included. We applied several statistical models and accounted effects of multiple covariates to provide more precise estimates of deciduous teeth dental caries over a long time period in Iran. Furthermore, our study uses rigorous methods to estimate dmft for each province in Iran. That has not been done previously in the literature. Our estimates cover sex and age subgroups, and most importantly, the estimates are presented by their 95% UIs.

### Limitations

While this study aimed to provide the most accurate data regarding the dmft of the Iranian children on national and sub-national scales, as all the entry data may not be fully accurate, the estimated results may not be adequately precise in some cases. This has been reflected in wide uncertainty intervals in some estimates. However, our results can satisfactorily inform oral health policies in the lack of accurate and transparent national child oral health surveys. This limitation remarkably highlights the need for improving the oral health national action plan (as part of the national health reform plan) for improving the oral health of the Iranian children. The first step should be regular national surveys to precisely estimate the prevalence of tooth decay among various provinces in Iran along with the incorporation of prevention methods.

## Conclusion

The trend of the child’s dental caries in Iran showed that high dmft in 1990 has increased continuously by more than 15%. Although the dmft was higher among boys compared to girls, however, this index has increases in all age groups, both sexes, and almost all provinces. This implies the urgent need to population-based interventions (such as supervised tooth brushing and improving dietary habits of this population) and changing the direction of oral health system policies. Although oral health was integrated in the national health reform plan since 2014 (which includes children under 14), however, studies with appropriate design to determine the cost-effectiveness of this plan are still lacking. This study’s results can be a backbone to inform oral health policy makers towards designing nationwide, transparent, and accurate oral health surveys and improving national oral health policies.

## Supplementary Information

**Additional file 1. Appendix 1.** Search strategy in each database. **Appendix 2.** Data extraction sheet’s content. **Appendix 3.** The used quality assessment tool based on the “STrengthening the Reporting of OBservational studies in Epidemiology” (STROBE). **Appendix 4.** Age-standardised deciduous teeth dmft and its components in subnational scale by sex. **Appendix 5.** Checklist of information that should be included in new reports of global health estimates

**Additional file 2.** Raw data.

## Data Availability

The data is available in the Supplementary materials (Additional File 2).

## Abbreviations

YLD: Years lived with disability
GBD: Global burden of disease
BOD: Burden of oral disorders
dmft: Decayed, missing, filled teeth in children
DT: Decayed teeth
MT: Missing teeth
FT: Filled teeth
GBD: Global burden of diseases study
ICD-10: International classification of diseases, 10th revision
MeSH: Medical subject headings
NASBOD: National and sub-national burden of diseases in Iran
SID: Scientific information database
STROBE: STrengthening the reporting of OBservational studies in epidemiology
UI: Uncertainty interval
WHO: World Health Organisation
