# Supplementary material for: Trends in dental caries of deciduous teeth in Iran: a systematic analysis of the national and sub-national data from 1990 to 2017
Source: BMC Oral Health. 2022 Dec 23;22:634. doi: 10.1186/s12903-022-02634-z (PMC9789600; doi:10.1186/s12903-022-02634-z)
Supplement: Supplementary file 1 — Additional file 1. Appendix 1. Search strategy in each database. Appendix 2. Data extraction sheet’s content. Appendix 3. The used quality assessment tool based on the “STrengthening the Reporting of OBservational studies in Epidemiology” (STROBE). Appendix 4. Age-standardised deciduous teeth dmft and its components in subnational scale by sex. Appendix 5. Checklist of information that should be included in new reports of global health estimates [file 12903_2022_2634_MOESM1_ESM.docx]

National and sub-national trend of dental caries in deciduous teeth in Iran: 1990-2017

Shervan Shoaee^1,2,3^, Sahar Saeedi Moghaddam^2^, Masoud Masinaei^2^, Ahmad Sofi-Mahmudi^2,4^, Hossein Hessari^5^, Erfan Shamsoddin^2,4^, Mohammad-Hossein Heydari^2,6^, Mahboubeh Parsaeian^7^, Anooshe Ghasemian^2^, Hossein Fakhrzadeh^8^, Bagher Larijani^8^, Farshad Farzadfar^2,8^

^1^ Elderly Health Research Center, Endocrinology and Metabolism Population Sciences Institute, Tehran University of Medical Sciences, Tehran, Iran.

^2^ Non-Communicable Diseases Research Center, Endocrinology and Metabolism Population Sciences Institute, Tehran University of Medical Sciences, Tehran, Iran.

^3^ Kerman Oral and Dental Diseases Research Center, Kerman University of Medical Sciences, Kerman, Iran.

^4^ Cochrane Iran Associate Centre, National Institute for Medical Research Development (NIMAD), Tehran, Iran.

^5^ Research Center for Caries Prevention, Dentistry Research Institute, Tehran University of Medical Sciences, Tehran, Iran.

^6^ School of Dentistry, Shahid Beh0eshti University of Medical Sciences, Tehran, Iran.

^7^ Department of Epidemiology and Biostatistics, School of Public Health, Tehran University of Medical Sciences, Tehran, Iran

^8^ Endocrinology and Metabolism Research Center, Endocrinology and Metabolism Clinical Sciences Institute, Tehran University of Medical Sciences, Tehran, Iran.

Corresponding authors

Farshad Farzadfar, MD, MPH, MHS, DSc

Non-Communicable Diseases Research Center, Endocrinology and Metabolism Population Sciences Institute, Tehran University of Medical Sciences, Tehran, Iran

Address: No. 10, Al-e-Ahmad and Chamran Highway intersection, Tehran, Iran

Postal code: 1411713136

Tel/Fax: +982188631293

E-mail: f-farzadfar@tums.ac.ir

Hossein Fakhrzadeh, MD

Endocrinology and Metabolism Research Center, Endocrinology and Metabolism Clinical Sciences Institute, Tehran University of Medical Sciences, Tehran, Iran

Address: No. 10, Al-e-Ahmad and Chamran Highway intersection, Tehran, Iran

Postal code: 1411713111

Tel/Fax: +982188220085

E-mail: fakhrzad@sina.tums.ac.ir

**Appendix 1**. Search strategy in each database

**Dental caries**

- PubMed:

(((("Dental Caries"[Mesh]) OR "DMF Index"[Mesh]) OR "Dental Restoration, Permanent"[Mesh]) OR "Tooth Diseases"[Mesh]) OR "Dental Health Surveys"[Mesh]

- ISI and Scopus:

“Dental Caries” OR “dental decay” OR “dental white spot” OR “DMF Index” OR “decayed, missing and filled teeth” OR “Dental Restoration, Permanent” OR “dental filling, permanent” OR “Tooth Diseases” OR “Dental Health Surveys”

- SID, IranMedex, and IranDoc

پوسیدگی دندان/ پوسیدگی های دندان/ پوسیدگیهای دندان/ شاخص دندانهای پوسیده افتاده و پرشده/ شاخص دندانهای پوسیده کشیده و پرشده/ شاخص دندانهای پوسیده از دست رفته و پرشده/ دندان های پوسیده افتاده و پرشده/ دندان های پوسیده کشیده و پرشده/ دندان های پوسیده از دست رفته و پرشده/ شاخص دی ام اف/ دی ام اف/ DMF /DMFشاخص / ترمیم دندانی/ ترمیم دائمی دندان/ بیماری های دندان/ بیماریهای دندان/ مطالعات بهداشت دندان

**Appendix 2**. Data extraction sheet’s content

| 1. Study characteristics information | |
| --- | --- |
| General information | Study ID: abbreviation of the disease, province code, and a number for each article |
|  | Study name: special name of national and sub-national study |
|  | Citation |
|  | Corresponding author’s characteristics |
|  | Article code (PMID or DOI) |
|  | Publication year |
|  | Study year |
|  | Practical definition: description of diseases |
|  | Subgroup: ethnicity of the study population |
|  | Study included/excluded by, and study cross-checked by (name of people) |
| Study source | 1. Iranian database |
|  | 1. International database |
|  | 1. Non-indexed Iranian journals |
|  | 1. Health surveys |
|  | 1. Presented abstract in a congress |
|  | 1. Theses |
|  | 1. Other unpublished reports |
| Study design | 1. Cross-sectional |
|  | 1. Case-control |
|  | 1. Cohort |
|  | 1. Others |
| Study scope | 1. Rural |
|  | 1. Urban |
|  | 1. Both |
| Study level | 1. National |
|  | 1. Provincial |
|  | 1. District |
|  | 1. Community |
|  | 1. Subregion: two or more provinces with similar ethnicity and race |
|  | 1. Two or more subregions |
| Sample weighting | 1. No |
|  | 1. Yes |
| Sampling | |
| Sampling method | 1. Multi-level clustering random sampling |
|  | 1. One-level clustering random sampling |
|  | 1. Simple random sampling |
|  | 1. Others |
| Sample size | 1. Less than 250 |
|  | 1. 250 – 500 |
|  | 1. 500 – 1000 |
|  | 1. More than 1000 |
| Response rate | 1. Less than 60% |
|  | 1. 60% – 74% |
|  | 1. 75% – 89% |
|  | 6. More than 90% |
| Quality assessment measurement | |
| Measurement | Type of measurement tools |
|  | Tools calibration |
|  | Validity and reliability of questionnaires, examiners, or instruments |
| 1. Data extraction information |  |
| Sex | Male |
|  | Female |
|  | Both |
| Age | Age start |
|  | Age end |
|  | Median age range |
| Sample size | Sample size in each sex and age group of the study population |
| Point prevalence or incidence | The point estimate, mean, SD, SE, lower and upper level of Cis are extracted from the included articles |
| Data extracted by, and cross-checked by | The name of people who will extract and cross-check the data |

**Appendix 3**. The used quality assessment tool based on the “STrengthening the Reporting of OBservational studies in Epidemiology” (STROBE)


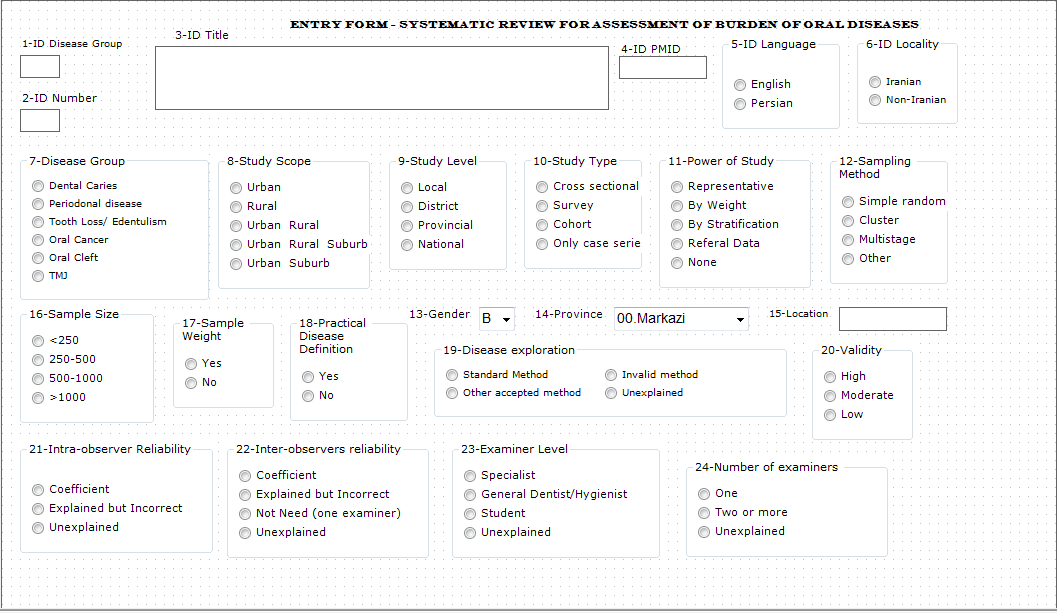


Appendix 4. Age-standardised deciduous teeth dmft and its components in subnational scale by sex

| Location | Year | dmft | | | dt | | | mt | | | ft | | |
| --- | --- | --- | --- | --- | --- | --- | --- | --- | --- | --- | --- | --- | --- |
|  |  | Both | Female | Male | Both | Female | Male | Both | Female | Male | Both | Female | Male |
| Alborz | 1990 | 4.05 (1.81, 6.37) | 3.72 (1.55, 6.06) | 4.36 (2.06, 6.66) | 3.38 (1.25, 5.65) | 3.18 (1.12, 5.47) | 3.56 (1.37, 5.82) | 0.35 (0.11, 0.73) | 0.28 (0.1, 0.63) | 0.41 (0.14, 0.82) | 0.33 (0.02, 0.69) | 0.27 (0, 0.62) | 0.39 (0.05, 0.75) |
|  | 2000 | 4.4 (2.07, 6.79) | 4.2 (1.86, 6.61) | 4.59 (2.27, 6.96) | 3.66 (1.29, 6.25) | 3.58 (1.23, 6.18) | 3.73 (1.34, 6.32) | 0.35 (0.11, 0.75) | 0.29 (0.1, 0.66) | 0.41 (0.13, 0.84) | 0.39 (0.03, 0.8) | 0.33 (0, 0.74) | 0.45 (0.06, 0.86) |
|  | 2010 | 4.5 (2.19, 6.88) | 4.41 (2.09, 6.8) | 4.58 (2.29, 6.95) | 3.74 (1.37, 6.21) | 3.73 (1.38, 6.18) | 3.74 (1.37, 6.24) | 0.34 (0.11, 0.73) | 0.3 (0.1, 0.66) | 0.38 (0.12, 0.79) | 0.42 (0.04, 0.82) | 0.38 (0.02, 0.77) | 0.47 (0.07, 0.87) |
|  | 2017 | 4.53 (2.32, 6.77) | 4.49 (2.29, 6.75) | 4.57 (2.35, 6.79) | 3.79 (1.52, 6.03) | 3.81 (1.56, 6.01) | 3.78 (1.49, 6.05) | 0.32 (0.1, 0.67) | 0.3 (0.1, 0.63) | 0.35 (0.11, 0.71) | 0.41 (0.06, 0.77) | 0.39 (0.04, 0.75) | 0.44 (0.07, 0.8) |
| Ardabil | 1990 | 5.3 (3.14, 7.53) | 5.01 (2.87, 7.24) | 5.58 (3.4, 7.8) | 4.75 (2.53, 6.99) | 4.48 (2.3, 6.73) | 4.99 (2.75, 7.24) | 0.44 (0.16, 0.84) | 0.41 (0.2, 0.8) | 0.47 (0.17, 0.88) | 0.11 (0, 0.47) | 0.12 (0, 0.47) | 0.11 (0, 0.47) |
|  | 2000 | 5.66 (3.41, 7.97) | 5.41 (3.17, 7.71) | 5.9 (3.64, 8.21) | 5.09 (2.63, 7.58) | 4.86 (2.38, 7.35) | 5.31 (2.86, 7.8) | 0.47 (0.17, 0.89) | 0.45 (0.2, 0.87) | 0.48 (0.17, 0.92) | 0.1 (0, 0.5) | 0.11 (0, 0.51) | 0.1 (0, 0.5) |
|  | 2010 | 5.8 (3.5, 8.16) | 5.64 (3.35, 8.01) | 5.94 (3.64, 8.3) | 5.2 (2.72, 7.69) | 5.06 (2.56, 7.54) | 5.33 (2.88, 7.83) | 0.48 (0.16, 0.91) | 0.48 (0.2, 0.91) | 0.49 (0.17, 0.92) | 0.11 (0, 0.51) | 0.1 (0, 0.5) | 0.12 (0, 0.52) |
|  | 2017 | 5.8 (3.55, 8.1) | 5.75 (3.49, 8.06) | 5.85 (3.61, 8.13) | 5.2 (2.81, 7.57) | 5.17 (2.77, 7.53) | 5.23 (2.85, 7.61) | 0.48 (0.16, 0.9) | 0.48 (0.2, 0.9) | 0.48 (0.16, 0.9) | 0.12 (0, 0.5) | 0.1 (0, 0.48) | 0.14 (0, 0.52) |
| Azerbaijan, East | 1990 | 5.2 (3, 7.44) | 4.83 (2.67, 7.05) | 5.54 (3.32, 7.8) | 4.51 (2.29, 6.69) | 4.21 (2.04, 6.39) | 4.79 (2.52, 6.99) | 0.49 (0.21, 0.9) | 0.42 (0.2, 0.8) | 0.55 (0.26, 0.99) | 0.2 (0, 0.54) | 0.2 (0, 0.54) | 0.2 (0, 0.55) |
|  | 2000 | 5.45 (3.15, 7.79) | 5.15 (2.84, 7.49) | 5.73 (3.43, 8.08) | 4.79 (2.29, 7.26) | 4.56 (2.06, 7.05) | 5 (2.51, 7.45) | 0.46 (0.17, 0.89) | 0.4 (0.1, 0.8) | 0.52 (0.21, 0.97) | 0.19 (0, 0.59) | 0.18 (0, 0.58) | 0.2 (0, 0.59) |
|  | 2010 | 5.4 (3.06, 7.78) | 5.2 (2.85, 7.59) | 5.59 (3.26, 7.97) | 4.76 (2.26, 7.2) | 4.62 (2.12, 7.08) | 4.88 (2.4, 7.32) | 0.43 (0.14, 0.84) | 0.38 (0.1, 0.77) | 0.48 (0.17, 0.91) | 0.21 (0, 0.61) | 0.2 (0, 0.59) | 0.23 (0, 0.62) |
|  | 2017 | 5.25 (3, 7.54) | 5.14 (2.9, 7.44) | 5.35 (3.1, 7.63) | 4.63 (2.27, 6.92) | 4.58 (2.21, 6.87) | 4.67 (2.33, 6.97) | 0.39 (0.13, 0.78) | 0.35 (0.1, 0.73) | 0.43 (0.14, 0.83) | 0.23 (0, 0.6) | 0.21 (0, 0.58) | 0.25 (0, 0.62) |
| Azerbaijan, West | 1990 | 4.95 (2.77, 7.18) | 4.54 (2.36, 6.76) | 5.34 (3.17, 7.58) | 4.4 (2.23, 6.58) | 4.01 (1.88, 6.2) | 4.77 (2.57, 6.95) | 0.38 (0.14, 0.76) | 0.33 (0.1, 0.69) | 0.43 (0.16, 0.82) | 0.17 (0, 0.51) | 0.19 (0, 0.54) | 0.14 (0, 0.48) |
|  | 2000 | 5.34 (3.09, 7.68) | 5.02 (2.78, 7.34) | 5.64 (3.39, 8) | 4.75 (2.32, 7.21) | 4.44 (2.05, 6.91) | 5.05 (2.58, 7.5) | 0.4 (0.14, 0.8) | 0.36 (0.1, 0.75) | 0.44 (0.15, 0.85) | 0.18 (0, 0.58) | 0.21 (0, 0.61) | 0.15 (0, 0.54) |
|  | 2010 | 5.56 (3.28, 7.94) | 5.36 (3.1, 7.72) | 5.74 (3.45, 8.15) | 4.94 (2.47, 7.4) | 4.74 (2.26, 7.19) | 5.14 (2.67, 7.6) | 0.42 (0.14, 0.82) | 0.4 (0.1, 0.79) | 0.44 (0.15, 0.85) | 0.19 (0, 0.58) | 0.22 (0, 0.61) | 0.17 (0, 0.56) |
|  | 2017 | 5.67 (3.45, 7.98) | 5.56 (3.35, 7.86) | 5.77 (3.54, 8.09) | 5.06 (2.71, 7.41) | 4.94 (2.57, 7.29) | 5.18 (2.84, 7.53) | 0.42 (0.13, 0.81) | 0.41 (0.1, 0.8) | 0.43 (0.14, 0.82) | 0.19 (0, 0.56) | 0.21 (0, 0.58) | 0.16 (0, 0.54) |
| Bushehr | 1990 | 2.91 (1.13, 5.16) | 2.83 (1.08, 5.04) | 2.99 (1.17, 5.27) | 2.53 (0.73, 4.74) | 2.43 (0.66, 4.66) | 2.62 (0.81, 4.81) | 0.23 (0.07, 0.55) | 0.23 (0.1, 0.55) | 0.23 (0.06, 0.55) | 0.16 (0, 0.51) | 0.18 (0, 0.53) | 0.14 (0, 0.49) |
|  | 2000 | 3.45 (1.43, 5.8) | 3.36 (1.34, 5.71) | 3.54 (1.5, 5.89) | 3.03 (0.86, 5.88) | 2.9 (0.73, 5.82) | 3.16 (0.99, 5.94) | 0.25 (0.07, 0.62) | 0.26 (0.1, 0.63) | 0.24 (0.07, 0.61) | 0.17 (0, 0.62) | 0.2 (0, 0.67) | 0.14 (0, 0.58) |
|  | 2010 | 3.76 (1.6, 6.14) | 3.68 (1.53, 6.05) | 3.83 (1.66, 6.22) | 3.3 (1.06, 5.95) | 3.16 (0.94, 5.86) | 3.42 (1.17, 6.03) | 0.28 (0.08, 0.65) | 0.3 (0.1, 0.67) | 0.27 (0.08, 0.62) | 0.18 (0, 0.61) | 0.22 (0, 0.65) | 0.14 (0, 0.57) |
|  | 2017 | 3.89 (1.73, 6.17) | 3.83 (1.68, 6.1) | 3.94 (1.77, 6.24) | 3.41 (1.25, 5.75) | 3.3 (1.16, 5.65) | 3.52 (1.33, 5.85) | 0.29 (0.08, 0.64) | 0.31 (0.1, 0.67) | 0.28 (0.07, 0.62) | 0.18 (0, 0.56) | 0.21 (0, 0.6) | 0.15 (0, 0.53) |
| Chahar Mahaal and Bakhtiari | 1990 | 4.69 (2.51, 6.92) | 4.39 (2.23, 6.61) | 4.97 (2.77, 7.21) | 3.94 (1.74, 6.19) | 3.68 (1.56, 5.96) | 4.18 (1.92, 6.41) | 0.54 (0.24, 0.96) | 0.48 (0.2, 0.9) | 0.6 (0.28, 1.03) | 0.21 (0, 0.57) | 0.23 (0, 0.59) | 0.2 (0, 0.55) |
|  | 2000 | 5.19 (2.9, 7.5) | 4.87 (2.59, 7.18) | 5.49 (3.2, 7.8) | 4.37 (1.9, 6.95) | 4.08 (1.67, 6.71) | 4.64 (2.11, 7.19) | 0.56 (0.24, 1.04) | 0.51 (0.2, 0.98) | 0.6 (0.28, 1.09) | 0.26 (0, 0.67) | 0.28 (0, 0.69) | 0.25 (0, 0.65) |
|  | 2010 | 5.44 (3.14, 7.78) | 5.16 (2.86, 7.5) | 5.71 (3.41, 8.05) | 4.55 (2.07, 7.07) | 4.32 (1.84, 6.88) | 4.77 (2.29, 7.25) | 0.56 (0.24, 1.03) | 0.53 (0.2, 0.99) | 0.59 (0.28, 1.07) | 0.33 (0, 0.73) | 0.31 (0, 0.72) | 0.34 (0.01, 0.73) |
|  | 2017 | 5.55 (3.31, 7.77) | 5.32 (3.08, 7.54) | 5.76 (3.54, 7.98) | 4.64 (2.29, 6.96) | 4.48 (2.11, 6.84) | 4.79 (2.46, 7.07) | 0.54 (0.24, 0.99) | 0.52 (0.2, 0.96) | 0.57 (0.27, 1.02) | 0.36 (0.02, 0.73) | 0.33 (0, 0.7) | 0.4 (0.04, 0.77) |
| Fars | 1990 | 4.48 (2.28, 6.74) | 4.24 (2.05, 6.5) | 4.7 (2.5, 6.97) | 3.82 (1.59, 6.1) | 3.6 (1.4, 5.89) | 4.04 (1.77, 6.3) | 0.46 (0.18, 0.88) | 0.43 (0.2, 0.84) | 0.49 (0.2, 0.91) | 0.19 (0, 0.55) | 0.21 (0, 0.57) | 0.17 (0, 0.54) |
|  | 2000 | 4.94 (2.66, 7.29) | 4.77 (2.5, 7.14) | 5.1 (2.81, 7.45) | 4.24 (1.69, 6.83) | 4.07 (1.55, 6.65) | 4.41 (1.82, 6.99) | 0.48 (0.17, 0.93) | 0.45 (0.2, 0.9) | 0.5 (0.18, 0.96) | 0.21 (0, 0.63) | 0.24 (0, 0.66) | 0.19 (0, 0.6) |
|  | 2010 | 5.13 (2.84, 7.52) | 5.05 (2.76, 7.44) | 5.21 (2.92, 7.6) | 4.42 (1.85, 6.94) | 4.32 (1.76, 6.85) | 4.51 (1.94, 7.02) | 0.48 (0.17, 0.92) | 0.47 (0.2, 0.91) | 0.49 (0.17, 0.94) | 0.24 (0, 0.64) | 0.27 (0, 0.67) | 0.21 (0, 0.61) |
|  | 2017 | 5.2 (2.99, 7.46) | 5.18 (2.96, 7.44) | 5.21 (3.02, 7.48) | 4.5 (2.1, 6.84) | 4.46 (2.06, 6.8) | 4.53 (2.14, 6.88) | 0.47 (0.16, 0.88) | 0.46 (0.2, 0.88) | 0.47 (0.16, 0.89) | 0.24 (0, 0.62) | 0.27 (0, 0.64) | 0.21 (0, 0.59) |
| Gilan | 1990 | 4 (1.91, 6.26) | 3.61 (1.65, 5.88) | 4.36 (2.15, 6.61) | 3.33 (1.29, 5.62) | 2.96 (1.08, 5.27) | 3.69 (1.48, 5.96) | 0.44 (0.17, 0.86) | 0.39 (0.1, 0.79) | 0.48 (0.2, 0.92) | 0.23 (0, 0.58) | 0.26 (0, 0.62) | 0.19 (0, 0.55) |
|  | 2000 | 4.56 (2.29, 6.88) | 4.21 (1.98, 6.52) | 4.89 (2.6, 7.21) | 3.84 (1.44, 6.52) | 3.48 (1.23, 6.26) | 4.17 (1.64, 6.77) | 0.47 (0.17, 0.94) | 0.44 (0.2, 0.9) | 0.5 (0.18, 0.98) | 0.25 (0, 0.67) | 0.28 (0, 0.72) | 0.22 (0, 0.63) |
|  | 2010 | 4.86 (2.54, 7.22) | 4.56 (2.23, 6.91) | 5.15 (2.83, 7.51) | 4.12 (1.67, 6.69) | 3.8 (1.46, 6.43) | 4.41 (1.88, 6.94) | 0.49 (0.17, 0.94) | 0.48 (0.2, 0.92) | 0.51 (0.18, 0.96) | 0.25 (0, 0.66) | 0.28 (0, 0.7) | 0.23 (0, 0.63) |
|  | 2017 | 5.01 (2.76, 7.28) | 4.73 (2.49, 7.01) | 5.27 (3.02, 7.54) | 4.28 (1.93, 6.63) | 3.98 (1.65, 6.33) | 4.55 (2.19, 6.9) | 0.49 (0.18, 0.92) | 0.48 (0.2, 0.91) | 0.5 (0.18, 0.93) | 0.24 (0, 0.62) | 0.26 (0, 0.65) | 0.22 (0, 0.6) |
| Golestan | 1990 | 4.53 (2.31, 6.77) | 4.27 (2.05, 6.5) | 4.77 (2.55, 7.02) | 4.01 (1.78, 6.26) | 3.79 (1.57, 5.99) | 4.23 (1.98, 6.5) | 0.38 (0.13, 0.75) | 0.32 (0.1, 0.68) | 0.43 (0.15, 0.82) | 0.14 (0, 0.49) | 0.16 (0, 0.5) | 0.12 (0, 0.48) |
|  | 2000 | 5.09 (2.8, 7.44) | 4.96 (2.67, 7.3) | 5.21 (2.91, 7.58) | 4.54 (2.03, 7.08) | 4.43 (1.9, 6.93) | 4.66 (2.15, 7.21) | 0.39 (0.13, 0.8) | 0.36 (0.1, 0.76) | 0.42 (0.15, 0.85) | 0.15 (0, 0.56) | 0.17 (0, 0.57) | 0.14 (0, 0.54) |
|  | 2010 | 5.53 (3.22, 7.9) | 5.48 (3.17, 7.86) | 5.57 (3.27, 7.94) | 4.96 (2.48, 7.41) | 4.89 (2.43, 7.32) | 5.02 (2.52, 7.49) | 0.4 (0.13, 0.8) | 0.39 (0.1, 0.79) | 0.41 (0.14, 0.81) | 0.17 (0, 0.56) | 0.2 (0, 0.58) | 0.14 (0, 0.53) |
|  | 2017 | 5.81 (3.6, 8.06) | 5.81 (3.58, 8.08) | 5.8 (3.63, 8.04) | 5.24 (2.93, 7.52) | 5.2 (2.88, 7.47) | 5.28 (2.97, 7.58) | 0.39 (0.12, 0.77) | 0.4 (0.1, 0.78) | 0.38 (0.12, 0.76) | 0.17 (0, 0.53) | 0.21 (0, 0.57) | 0.14 (0, 0.5) |
| Hamadan | 1990 | 4.1 (1.97, 6.37) | 4.04 (1.88, 6.29) | 4.17 (2.06, 6.45) | 3.54 (1.44, 5.73) | 3.46 (1.35, 5.63) | 3.6 (1.54, 5.83) | 0.38 (0.14, 0.77) | 0.37 (0.1, 0.74) | 0.4 (0.14, 0.79) | 0.19 (0, 0.54) | 0.21 (0, 0.56) | 0.17 (0, 0.53) |
|  | 2000 | 4.67 (2.4, 7.02) | 4.64 (2.37, 6.97) | 4.7 (2.42, 7.06) | 4.06 (1.61, 6.58) | 4.02 (1.53, 6.51) | 4.09 (1.7, 6.65) | 0.42 (0.14, 0.83) | 0.4 (0.1, 0.81) | 0.43 (0.15, 0.86) | 0.2 (0, 0.61) | 0.21 (0, 0.63) | 0.18 (0, 0.6) |
|  | 2010 | 5.02 (2.72, 7.38) | 5.01 (2.71, 7.37) | 5.03 (2.74, 7.39) | 4.37 (1.83, 6.84) | 4.34 (1.79, 6.8) | 4.39 (1.88, 6.88) | 0.43 (0.15, 0.85) | 0.43 (0.1, 0.83) | 0.44 (0.15, 0.86) | 0.22 (0, 0.62) | 0.24 (0, 0.64) | 0.2 (0, 0.6) |
|  | 2017 | 5.2 (2.97, 7.45) | 5.18 (2.94, 7.46) | 5.21 (3, 7.45) | 4.54 (2.16, 6.85) | 4.51 (2.13, 6.8) | 4.57 (2.18, 6.9) | 0.43 (0.14, 0.83) | 0.43 (0.1, 0.82) | 0.44 (0.15, 0.84) | 0.23 (0, 0.6) | 0.25 (0, 0.62) | 0.21 (0, 0.58) |
| Hormozgan | 1990 | 2.95 (1.15, 5.2) | 2.69 (0.96, 4.96) | 3.18 (1.34, 5.43) | 2.53 (0.78, 4.73) | 2.29 (0.59, 4.47) | 2.76 (0.96, 4.98) | 0.24 (0.08, 0.59) | 0.24 (0.1, 0.58) | 0.25 (0.08, 0.59) | 0.17 (0, 0.52) | 0.17 (0, 0.5) | 0.18 (0, 0.53) |
|  | 2000 | 3.66 (1.57, 6.01) | 3.48 (1.43, 5.81) | 3.83 (1.7, 6.21) | 3.24 (1.04, 6.06) | 3.06 (0.9, 5.93) | 3.41 (1.17, 6.18) | 0.28 (0.09, 0.68) | 0.28 (0.1, 0.67) | 0.29 (0.09, 0.68) | 0.14 (0, 0.58) | 0.14 (0, 0.59) | 0.14 (0, 0.57) |
|  | 2010 | 4.16 (1.87, 6.55) | 4.05 (1.79, 6.43) | 4.27 (1.95, 6.68) | 3.74 (1.39, 6.38) | 3.62 (1.3, 6.28) | 3.85 (1.48, 6.47) | 0.32 (0.1, 0.7) | 0.32 (0.1, 0.7) | 0.32 (0.1, 0.71) | 0.11 (0, 0.52) | 0.11 (0, 0.53) | 0.1 (0, 0.51) |
|  | 2017 | 4.4 (2.14, 6.71) | 4.34 (2.07, 6.65) | 4.46 (2.21, 6.76) | 3.97 (1.63, 6.36) | 3.9 (1.59, 6.3) | 4.03 (1.68, 6.41) | 0.33 (0.1, 0.7) | 0.33 (0.1, 0.7) | 0.33 (0.1, 0.7) | 0.1 (0, 0.48) | 0.11 (0, 0.49) | 0.09 (0, 0.47) |
| Ilam | 1990 | 4.86 (2.74, 7.1) | 4.25 (2.2, 6.46) | 5.44 (3.25, 7.71) | 4.06 (1.93, 6.35) | 3.54 (1.54, 5.92) | 4.55 (2.31, 6.77) | 0.68 (0.35, 1.12) | 0.58 (0.3, 1.03) | 0.78 (0.43, 1.2) | 0.12 (0, 0.48) | 0.13 (0, 0.5) | 0.11 (0, 0.47) |
|  | 2000 | 5.32 (3.06, 7.65) | 4.8 (2.53, 7.13) | 5.81 (3.56, 8.15) | 4.52 (2.08, 7.14) | 4.07 (1.69, 6.82) | 4.95 (2.45, 7.44) | 0.68 (0.34, 1.17) | 0.6 (0.3, 1.12) | 0.75 (0.39, 1.23) | 0.12 (0, 0.54) | 0.13 (0, 0.57) | 0.11 (0, 0.51) |
|  | 2010 | 5.55 (3.27, 7.92) | 5.18 (2.88, 7.55) | 5.91 (3.64, 8.28) | 4.79 (2.29, 7.33) | 4.46 (1.95, 7.07) | 5.11 (2.6, 7.58) | 0.64 (0.3, 1.12) | 0.59 (0.3, 1.08) | 0.69 (0.34, 1.16) | 0.12 (0, 0.53) | 0.13 (0, 0.55) | 0.12 (0, 0.51) |
|  | 2017 | 5.66 (3.46, 7.93) | 5.41 (3.2, 7.67) | 5.89 (3.71, 8.17) | 4.94 (2.55, 7.31) | 4.72 (2.31, 7.13) | 5.15 (2.77, 7.47) | 0.59 (0.27, 1.04) | 0.56 (0.2, 1.01) | 0.62 (0.3, 1.07) | 0.12 (0, 0.49) | 0.12 (0, 0.5) | 0.11 (0, 0.49) |
| Isfahan | 1990 | 4.23 (2.11, 6.52) | 3.89 (1.84, 6.19) | 4.55 (2.36, 6.83) | 3.28 (1.21, 5.5) | 2.95 (0.97, 5.22) | 3.59 (1.43, 5.77) | 0.53 (0.22, 0.96) | 0.47 (0.2, 0.91) | 0.58 (0.26, 1.01) | 0.42 (0.07, 0.78) | 0.47 (0.1, 0.83) | 0.38 (0.04, 0.73) |
|  | 2000 | 4.71 (2.44, 7.1) | 4.44 (2.16, 6.83) | 4.97 (2.72, 7.35) | 3.62 (1.26, 6.19) | 3.31 (1.06, 5.94) | 3.91 (1.45, 6.42) | 0.55 (0.22, 1.04) | 0.51 (0.2, 0.99) | 0.58 (0.25, 1.09) | 0.55 (0.13, 0.97) | 0.62 (0.19, 1.05) | 0.48 (0.07, 0.89) |
|  | 2010 | 4.95 (2.66, 7.36) | 4.75 (2.45, 7.17) | 5.14 (2.85, 7.54) | 3.76 (1.36, 6.26) | 3.51 (1.23, 6.04) | 4 (1.49, 6.46) | 0.55 (0.22, 1.03) | 0.52 (0.2, 1) | 0.58 (0.25, 1.07) | 0.64 (0.23, 1.05) | 0.72 (0.3, 1.14) | 0.56 (0.16, 0.97) |
|  | 2017 | 5.05 (2.83, 7.34) | 4.91 (2.69, 7.2) | 5.17 (2.96, 7.47) | 3.84 (1.49, 6.16) | 3.65 (1.38, 5.99) | 4.03 (1.59, 6.32) | 0.53 (0.22, 0.99) | 0.51 (0.2, 0.97) | 0.55 (0.24, 1.01) | 0.67 (0.29, 1.05) | 0.74 (0.36, 1.13) | 0.59 (0.22, 0.97) |
| Kerman | 1990 | 4.31 (2.1, 6.57) | 4.4 (2.16, 6.63) | 4.22 (2.04, 6.51) | 3.68 (1.52, 5.94) | 3.79 (1.54, 6.03) | 3.58 (1.51, 5.85) | 0.4 (0.14, 0.8) | 0.4 (0.1, 0.8) | 0.41 (0.14, 0.8) | 0.22 (0, 0.57) | 0.21 (0, 0.56) | 0.23 (0, 0.59) |
|  | 2000 | 4.94 (2.66, 7.29) | 5.01 (2.72, 7.36) | 4.88 (2.6, 7.22) | 4.32 (1.84, 6.91) | 4.42 (1.87, 6.99) | 4.22 (1.82, 6.84) | 0.42 (0.15, 0.85) | 0.41 (0.1, 0.84) | 0.43 (0.15, 0.86) | 0.21 (0, 0.61) | 0.19 (0, 0.59) | 0.22 (0, 0.63) |
|  | 2010 | 5.37 (3.09, 7.72) | 5.44 (3.16, 7.78) | 5.3 (3.03, 7.65) | 4.74 (2.25, 7.26) | 4.84 (2.36, 7.35) | 4.64 (2.15, 7.18) | 0.44 (0.15, 0.86) | 0.43 (0.1, 0.85) | 0.45 (0.15, 0.87) | 0.19 (0, 0.58) | 0.17 (0, 0.56) | 0.21 (0, 0.61) |
|  | 2017 | 5.58 (3.39, 7.8) | 5.66 (3.46, 7.87) | 5.5 (3.33, 7.74) | 4.95 (2.64, 7.28) | 5.05 (2.74, 7.37) | 4.85 (2.55, 7.2) | 0.44 (0.15, 0.84) | 0.43 (0.1, 0.83) | 0.45 (0.15, 0.85) | 0.19 (0, 0.55) | 0.17 (0, 0.53) | 0.21 (0, 0.57) |
| Kermanshah | 1990 | 4.42 (2.3, 6.66) | 4.25 (2.2, 6.46) | 4.57 (2.4, 6.84) | 3.75 (1.69, 5.98) | 3.6 (1.64, 5.85) | 3.89 (1.73, 6.11) | 0.48 (0.2, 0.88) | 0.45 (0.2, 0.85) | 0.5 (0.22, 0.91) | 0.19 (0, 0.54) | 0.2 (0, 0.55) | 0.19 (0, 0.54) |
|  | 2000 | 4.85 (2.59, 7.17) | 4.69 (2.44, 7) | 5 (2.73, 7.34) | 4.14 (1.8, 6.72) | 4 (1.74, 6.61) | 4.27 (1.86, 6.81) | 0.5 (0.19, 0.95) | 0.47 (0.2, 0.92) | 0.53 (0.21, 0.99) | 0.21 (0, 0.62) | 0.21 (0, 0.63) | 0.21 (0, 0.62) |
|  | 2010 | 5.06 (2.77, 7.44) | 4.95 (2.66, 7.33) | 5.18 (2.88, 7.54) | 4.34 (1.91, 6.85) | 4.25 (1.87, 6.75) | 4.44 (1.94, 6.93) | 0.5 (0.19, 0.94) | 0.48 (0.2, 0.91) | 0.52 (0.21, 0.97) | 0.22 (0, 0.62) | 0.22 (0, 0.62) | 0.22 (0, 0.62) |
|  | 2017 | 5.12 (2.9, 7.4) | 5.05 (2.82, 7.34) | 5.18 (2.97, 7.46) | 4.42 (2.08, 6.73) | 4.37 (2.04, 6.69) | 4.47 (2.12, 6.77) | 0.48 (0.18, 0.89) | 0.47 (0.2, 0.87) | 0.49 (0.2, 0.92) | 0.22 (0, 0.59) | 0.22 (0, 0.59) | 0.22 (0, 0.59) |
| Khorasan, North | 1990 | 3.99 (1.88, 6.21) | 3.85 (1.82, 6.06) | 4.11 (1.93, 6.36) | 3.54 (1.46, 5.8) | 3.41 (1.41, 5.66) | 3.66 (1.52, 5.92) | 0.27 (0.09, 0.62) | 0.25 (0.1, 0.58) | 0.3 (0.1, 0.65) | 0.17 (0, 0.52) | 0.2 (0, 0.54) | 0.15 (0, 0.51) |
|  | 2000 | 4.43 (2.15, 6.76) | 4.27 (2.02, 6.6) | 4.58 (2.28, 6.92) | 3.96 (1.55, 6.62) | 3.8 (1.46, 6.47) | 4.11 (1.63, 6.76) | 0.32 (0.1, 0.7) | 0.3 (0.1, 0.68) | 0.34 (0.11, 0.73) | 0.15 (0, 0.57) | 0.17 (0, 0.59) | 0.13 (0, 0.55) |
|  | 2010 | 4.59 (2.27, 6.95) | 4.42 (2.11, 6.81) | 4.74 (2.43, 7.09) | 4.08 (1.61, 6.67) | 3.92 (1.52, 6.53) | 4.24 (1.7, 6.81) | 0.36 (0.11, 0.76) | 0.35 (0.1, 0.75) | 0.37 (0.12, 0.77) | 0.14 (0, 0.55) | 0.16 (0, 0.57) | 0.13 (0, 0.54) |
|  | 2017 | 4.61 (2.39, 6.88) | 4.47 (2.23, 6.76) | 4.74 (2.54, 6.99) | 4.1 (1.74, 6.47) | 3.96 (1.62, 6.36) | 4.24 (1.86, 6.58) | 0.37 (0.11, 0.75) | 0.37 (0.1, 0.75) | 0.38 (0.11, 0.76) | 0.13 (0, 0.51) | 0.14 (0, 0.52) | 0.13 (0, 0.5) |
| Khorasan, Razavi | 1990 | 3.81 (1.74, 6.03) | 3.76 (1.73, 5.96) | 3.85 (1.75, 6.08) | 2.93 (1.05, 5.14) | 2.91 (1.06, 5.15) | 2.95 (1.03, 5.14) | 0.44 (0.16, 0.84) | 0.44 (0.2, 0.84) | 0.44 (0.16, 0.85) | 0.43 (0.09, 0.78) | 0.41 (0.08, 0.75) | 0.46 (0.11, 0.8) |
|  | 2000 | 4.22 (1.96, 6.57) | 4.17 (1.94, 6.5) | 4.28 (1.98, 6.62) | 3.26 (1.09, 5.92) | 3.23 (1.08, 5.96) | 3.29 (1.1, 5.89) | 0.49 (0.18, 0.96) | 0.5 (0.2, 0.97) | 0.49 (0.17, 0.95) | 0.47 (0.07, 0.89) | 0.44 (0.05, 0.87) | 0.5 (0.09, 0.91) |
|  | 2010 | 4.47 (2.17, 6.84) | 4.44 (2.14, 6.82) | 4.5 (2.2, 6.86) | 3.54 (1.27, 6.11) | 3.53 (1.28, 6.15) | 3.54 (1.26, 6.07) | 0.5 (0.19, 0.96) | 0.51 (0.2, 0.98) | 0.49 (0.18, 0.94) | 0.43 (0.04, 0.84) | 0.4 (0.02, 0.82) | 0.47 (0.06, 0.87) |
|  | 2017 | 4.64 (2.44, 6.89) | 4.65 (2.43, 6.91) | 4.63 (2.45, 6.87) | 3.79 (1.51, 6.12) | 3.82 (1.54, 6.16) | 3.76 (1.48, 6.07) | 0.48 (0.18, 0.91) | 0.5 (0.2, 0.93) | 0.47 (0.17, 0.89) | 0.37 (0.02, 0.74) | 0.33 (0.01, 0.71) | 0.4 (0.03, 0.77) |
| Khorasan, South | 1990 | 4.4 (2.18, 6.65) | 4.19 (2.03, 6.39) | 4.6 (2.33, 6.9) | 3.87 (1.67, 6.19) | 3.68 (1.57, 5.98) | 4.05 (1.77, 6.4) | 0.36 (0.13, 0.74) | 0.34 (0.1, 0.71) | 0.38 (0.13, 0.76) | 0.17 (0, 0.53) | 0.17 (0, 0.53) | 0.17 (0, 0.53) |
|  | 2000 | 4.92 (2.63, 7.27) | 4.75 (2.46, 7.09) | 5.08 (2.79, 7.43) | 4.38 (1.82, 7.03) | 4.23 (1.73, 6.93) | 4.52 (1.91, 7.12) | 0.4 (0.14, 0.81) | 0.39 (0.1, 0.8) | 0.41 (0.14, 0.81) | 0.14 (0, 0.57) | 0.13 (0, 0.57) | 0.15 (0, 0.57) |
|  | 2010 | 5.15 (2.84, 7.52) | 5.01 (2.68, 7.4) | 5.28 (2.99, 7.64) | 4.57 (1.98, 7.14) | 4.45 (1.8, 7.07) | 4.69 (2.14, 7.2) | 0.42 (0.14, 0.83) | 0.42 (0.1, 0.84) | 0.42 (0.14, 0.82) | 0.15 (0, 0.57) | 0.14 (0, 0.56) | 0.17 (0, 0.58) |
|  | 2017 | 5.2 (2.98, 7.47) | 5.08 (2.82, 7.37) | 5.32 (3.13, 7.56) | 4.62 (2.21, 6.97) | 4.51 (2.04, 6.89) | 4.72 (2.36, 7.04) | 0.42 (0.13, 0.81) | 0.43 (0.1, 0.82) | 0.41 (0.13, 0.8) | 0.16 (0, 0.55) | 0.14 (0, 0.54) | 0.18 (0, 0.55) |
| Khuzestan | 1990 | 3.9 (1.81, 6.16) | 3.77 (1.78, 6.01) | 4.03 (1.85, 6.31) | 3.35 (1.34, 5.52) | 3.2 (1.31, 5.37) | 3.49 (1.36, 5.67) | 0.31 (0.11, 0.66) | 0.29 (0.1, 0.62) | 0.34 (0.11, 0.7) | 0.24 (0, 0.58) | 0.28 (0, 0.62) | 0.2 (0, 0.54) |
|  | 2000 | 4.42 (2.18, 6.75) | 4.27 (2.06, 6.59) | 4.56 (2.29, 6.91) | 3.86 (1.51, 6.39) | 3.69 (1.46, 6.27) | 4.03 (1.56, 6.5) | 0.32 (0.1, 0.69) | 0.3 (0.1, 0.66) | 0.34 (0.11, 0.71) | 0.24 (0, 0.64) | 0.28 (0, 0.7) | 0.19 (0, 0.58) |
|  | 2010 | 4.74 (2.46, 7.11) | 4.61 (2.34, 6.97) | 4.86 (2.56, 7.23) | 4.2 (1.79, 6.66) | 4.06 (1.68, 6.56) | 4.34 (1.9, 6.76) | 0.32 (0.1, 0.69) | 0.31 (0.1, 0.67) | 0.34 (0.11, 0.71) | 0.21 (0, 0.6) | 0.24 (0, 0.64) | 0.18 (0, 0.57) |
|  | 2017 | 4.91 (2.7, 7.18) | 4.82 (2.61, 7.11) | 4.99 (2.78, 7.25) | 4.41 (2.1, 6.7) | 4.33 (1.99, 6.64) | 4.49 (2.2, 6.75) | 0.32 (0.09, 0.67) | 0.31 (0.1, 0.66) | 0.34 (0.1, 0.69) | 0.17 (0, 0.54) | 0.18 (0, 0.55) | 0.16 (0, 0.53) |
| Kohgiluyeh and Boyer-Ahmad | 1990 | 4.89 (2.69, 7.13) | 4.52 (2.35, 6.75) | 5.25 (3.02, 7.49) | 4.24 (2.09, 6.52) | 3.8 (1.73, 6.04) | 4.66 (2.43, 6.98) | 0.46 (0.17, 0.88) | 0.47 (0.2, 0.88) | 0.46 (0.16, 0.87) | 0.19 (0, 0.54) | 0.24 (0, 0.6) | 0.13 (0, 0.49) |
|  | 2000 | 5.31 (3.02, 7.65) | 4.98 (2.71, 7.31) | 5.63 (3.32, 7.98) | 4.66 (2.2, 7.22) | 4.27 (1.84, 6.88) | 5.02 (2.54, 7.54) | 0.49 (0.17, 0.93) | 0.49 (0.2, 0.95) | 0.48 (0.17, 0.92) | 0.17 (0, 0.58) | 0.22 (0, 0.64) | 0.13 (0, 0.52) |
|  | 2010 | 5.51 (3.19, 7.88) | 5.25 (2.94, 7.63) | 5.74 (3.43, 8.11) | 4.82 (2.31, 7.34) | 4.53 (2, 7.11) | 5.1 (2.61, 7.56) | 0.5 (0.18, 0.95) | 0.51 (0.2, 0.96) | 0.49 (0.17, 0.93) | 0.18 (0, 0.58) | 0.21 (0, 0.62) | 0.16 (0, 0.55) |
|  | 2017 | 5.57 (3.33, 7.83) | 5.4 (3.13, 7.65) | 5.73 (3.51, 8) | 4.89 (2.52, 7.25) | 4.69 (2.29, 7.1) | 5.08 (2.74, 7.39) | 0.49 (0.18, 0.93) | 0.51 (0.2, 0.94) | 0.48 (0.18, 0.91) | 0.19 (0, 0.56) | 0.21 (0, 0.58) | 0.17 (0, 0.54) |
| Kurdistan | 1990 | 4.87 (2.69, 7.09) | 4.75 (2.6, 6.95) | 4.98 (2.78, 7.22) | 4.22 (2.05, 6.47) | 4.14 (1.9, 6.51) | 4.29 (2.19, 6.42) | 0.52 (0.22, 0.94) | 0.47 (0.2, 0.9) | 0.57 (0.25, 0.98) | 0.13 (0, 0.48) | 0.15 (0, 0.52) | 0.11 (0, 0.45) |
|  | 2000 | 5.42 (3.17, 7.75) | 5.21 (2.96, 7.52) | 5.62 (3.36, 7.97) | 4.76 (2.3, 7.27) | 4.59 (2.09, 7.17) | 4.93 (2.49, 7.37) | 0.55 (0.22, 1.02) | 0.5 (0.2, 0.96) | 0.6 (0.26, 1.07) | 0.1 (0, 0.5) | 0.12 (0, 0.53) | 0.09 (0, 0.48) |
|  | 2010 | 5.81 (3.51, 8.17) | 5.56 (3.25, 7.92) | 6.04 (3.75, 8.41) | 5.15 (2.67, 7.6) | 4.94 (2.45, 7.4) | 5.35 (2.89, 7.79) | 0.55 (0.22, 1.02) | 0.51 (0.2, 0.97) | 0.59 (0.25, 1.07) | 0.1 (0, 0.5) | 0.11 (0, 0.51) | 0.1 (0, 0.49) |
|  | 2017 | 6.03 (3.78, 8.29) | 5.78 (3.5, 8.06) | 6.26 (4.04, 8.5) | 5.39 (3.04, 7.69) | 5.17 (2.83, 7.48) | 5.6 (3.24, 7.88) | 0.53 (0.21, 0.98) | 0.51 (0.2, 0.94) | 0.56 (0.23, 1.01) | 0.1 (0, 0.48) | 0.11 (0, 0.48) | 0.1 (0, 0.47) |
| Lorestan | 1990 | 5.04 (2.85, 7.24) | 4.83 (2.66, 7.02) | 5.24 (3.04, 7.45) | 4.32 (2.05, 6.6) | 4.17 (1.91, 6.43) | 4.46 (2.18, 6.75) | 0.57 (0.27, 1.01) | 0.52 (0.2, 0.96) | 0.63 (0.3, 1.07) | 0.14 (0, 0.5) | 0.14 (0, 0.49) | 0.15 (0, 0.5) |
|  | 2000 | 5.46 (3.17, 7.79) | 5.29 (2.99, 7.6) | 5.62 (3.33, 7.96) | 4.77 (2.17, 7.38) | 4.64 (2.03, 7.25) | 4.9 (2.3, 7.5) | 0.57 (0.25, 1.07) | 0.54 (0.2, 1.02) | 0.61 (0.28, 1.11) | 0.11 (0, 0.52) | 0.11 (0, 0.52) | 0.11 (0, 0.52) |
|  | 2010 | 5.58 (3.25, 7.94) | 5.49 (3.13, 7.86) | 5.67 (3.35, 8.02) | 4.9 (2.33, 7.47) | 4.83 (2.25, 7.41) | 4.97 (2.41, 7.52) | 0.56 (0.23, 1.05) | 0.54 (0.2, 1.02) | 0.58 (0.25, 1.07) | 0.12 (0, 0.52) | 0.12 (0, 0.52) | 0.12 (0, 0.52) |
|  | 2017 | 5.55 (3.29, 7.82) | 5.54 (3.23, 7.8) | 5.57 (3.34, 7.83) | 4.88 (2.49, 7.29) | 4.89 (2.48, 7.3) | 4.88 (2.5, 7.28) | 0.53 (0.21, 1) | 0.53 (0.2, 0.98) | 0.54 (0.23, 1.01) | 0.13 (0, 0.51) | 0.13 (0, 0.51) | 0.14 (0, 0.51) |
| Markazi | 1990 | 3.78 (1.74, 6.03) | 3.53 (1.56, 5.79) | 4.02 (1.91, 6.26) | 3.39 (1.39, 5.62) | 3.18 (1.23, 5.38) | 3.59 (1.55, 5.86) | 0.25 (0.08, 0.58) | 0.22 (0.1, 0.53) | 0.28 (0.09, 0.63) | 0.14 (0, 0.5) | 0.13 (0, 0.49) | 0.15 (0, 0.51) |
|  | 2000 | 4.28 (2.01, 6.63) | 4.11 (1.89, 6.48) | 4.43 (2.11, 6.78) | 3.84 (1.51, 6.47) | 3.71 (1.4, 6.37) | 3.97 (1.61, 6.57) | 0.27 (0.08, 0.64) | 0.25 (0.1, 0.6) | 0.3 (0.09, 0.67) | 0.16 (0, 0.59) | 0.16 (0, 0.59) | 0.16 (0, 0.59) |
|  | 2010 | 4.52 (2.18, 6.91) | 4.45 (2.11, 6.85) | 4.59 (2.26, 6.97) | 4.01 (1.63, 6.54) | 3.94 (1.58, 6.52) | 4.07 (1.67, 6.57) | 0.3 (0.09, 0.67) | 0.29 (0.1, 0.65) | 0.32 (0.1, 0.69) | 0.21 (0, 0.62) | 0.22 (0, 0.63) | 0.2 (0, 0.61) |
|  | 2017 | 4.59 (2.34, 6.89) | 4.57 (2.31, 6.88) | 4.6 (2.37, 6.89) | 4.03 (1.72, 6.35) | 4.01 (1.71, 6.35) | 4.05 (1.72, 6.34) | 0.32 (0.09, 0.66) | 0.31 (0.1, 0.65) | 0.33 (0.1, 0.68) | 0.24 (0, 0.61) | 0.26 (0, 0.64) | 0.22 (0, 0.59) |
| Mazandaran | 1990 | 4.46 (2.24, 6.73) | 4.29 (2.06, 6.54) | 4.62 (2.41, 6.91) | 3.7 (1.53, 5.96) | 3.61 (1.49, 5.84) | 3.78 (1.57, 6.06) | 0.51 (0.21, 0.93) | 0.44 (0.2, 0.84) | 0.59 (0.26, 1.01) | 0.25 (0, 0.6) | 0.24 (0, 0.59) | 0.25 (0, 0.61) |
|  | 2000 | 4.89 (2.6, 7.27) | 4.76 (2.46, 7.14) | 5.02 (2.72, 7.38) | 4.09 (1.64, 6.63) | 4.02 (1.61, 6.55) | 4.16 (1.67, 6.72) | 0.52 (0.2, 0.99) | 0.47 (0.2, 0.92) | 0.56 (0.24, 1.05) | 0.28 (0, 0.68) | 0.27 (0, 0.67) | 0.29 (0, 0.7) |
|  | 2010 | 5.05 (2.74, 7.43) | 4.96 (2.65, 7.35) | 5.13 (2.81, 7.5) | 4.22 (1.81, 6.63) | 4.16 (1.76, 6.56) | 4.27 (1.86, 6.69) | 0.5 (0.19, 0.95) | 0.48 (0.2, 0.92) | 0.52 (0.21, 0.98) | 0.33 (0, 0.71) | 0.31 (0, 0.69) | 0.34 (0, 0.72) |
|  | 2017 | 5.07 (2.84, 7.33) | 5.01 (2.81, 7.3) | 5.12 (2.88, 7.36) | 4.25 (2.05, 6.41) | 4.21 (2, 6.37) | 4.29 (2.1, 6.45) | 0.47 (0.19, 0.89) | 0.47 (0.2, 0.88) | 0.47 (0.19, 0.89) | 0.34 (0.02, 0.69) | 0.33 (0.01, 0.68) | 0.36 (0.03, 0.7) |
| Qazvin | 1990 | 3.6 (1.6, 5.86) | 3.34 (1.43, 5.6) | 3.85 (1.76, 6.11) | 3.1 (1.1, 5.36) | 2.89 (0.99, 5.17) | 3.3 (1.2, 5.55) | 0.33 (0.11, 0.71) | 0.28 (0.1, 0.63) | 0.38 (0.13, 0.78) | 0.17 (0, 0.53) | 0.17 (0, 0.53) | 0.16 (0, 0.52) |
|  | 2000 | 4.07 (1.91, 6.44) | 3.86 (1.76, 6.23) | 4.28 (2.06, 6.64) | 3.55 (1.24, 6.25) | 3.37 (1.15, 6.12) | 3.71 (1.33, 6.37) | 0.35 (0.11, 0.76) | 0.31 (0.1, 0.7) | 0.39 (0.13, 0.82) | 0.18 (0, 0.61) | 0.18 (0, 0.62) | 0.18 (0, 0.6) |
|  | 2010 | 4.33 (2.1, 6.7) | 4.18 (1.97, 6.54) | 4.48 (2.23, 6.85) | 3.79 (1.43, 6.35) | 3.66 (1.38, 6.23) | 3.91 (1.48, 6.46) | 0.36 (0.12, 0.76) | 0.33 (0.1, 0.72) | 0.39 (0.13, 0.79) | 0.19 (0, 0.6) | 0.19 (0, 0.61) | 0.19 (0, 0.6) |
|  | 2017 | 4.44 (2.28, 6.69) | 4.33 (2.17, 6.6) | 4.54 (2.37, 6.78) | 3.9 (1.59, 6.21) | 3.81 (1.57, 6.12) | 3.99 (1.61, 6.29) | 0.35 (0.11, 0.72) | 0.34 (0.1, 0.7) | 0.37 (0.12, 0.74) | 0.19 (0, 0.56) | 0.19 (0, 0.56) | 0.19 (0, 0.56) |
| Qom | 1990 | 4.01 (1.85, 6.27) | 3.77 (1.65, 6.05) | 4.24 (2.03, 6.47) | 3.53 (1.34, 5.81) | 3.3 (1.2, 5.6) | 3.75 (1.47, 6.01) | 0.32 (0.1, 0.7) | 0.32 (0.1, 0.71) | 0.33 (0.1, 0.7) | 0.16 (0, 0.52) | 0.16 (0, 0.52) | 0.16 (0, 0.52) |
|  | 2000 | 4.66 (2.35, 6.99) | 4.42 (2.1, 6.75) | 4.89 (2.58, 7.21) | 4.11 (1.63, 6.7) | 3.87 (1.44, 6.51) | 4.35 (1.81, 6.87) | 0.34 (0.11, 0.76) | 0.34 (0.1, 0.77) | 0.34 (0.11, 0.74) | 0.2 (0, 0.61) | 0.21 (0, 0.63) | 0.19 (0, 0.59) |
|  | 2010 | 5.09 (2.79, 7.43) | 4.85 (2.55, 7.18) | 5.31 (3.02, 7.66) | 4.47 (2.03, 6.94) | 4.21 (1.74, 6.73) | 4.72 (2.3, 7.14) | 0.36 (0.12, 0.76) | 0.37 (0.1, 0.78) | 0.36 (0.12, 0.74) | 0.25 (0, 0.64) | 0.27 (0, 0.67) | 0.24 (0, 0.62) |
|  | 2017 | 5.33 (3.15, 7.55) | 5.09 (2.92, 7.32) | 5.56 (3.37, 7.77) | 4.69 (2.42, 6.94) | 4.42 (2.11, 6.71) | 4.95 (2.72, 7.17) | 0.36 (0.13, 0.74) | 0.37 (0.1, 0.76) | 0.36 (0.13, 0.72) | 0.28 (0, 0.63) | 0.3 (0, 0.66) | 0.25 (0, 0.6) |
| Semnan | 1990 | 4.09 (1.94, 6.36) | 3.81 (1.72, 6.05) | 4.36 (2.15, 6.66) | 3.55 (1.41, 5.78) | 3.29 (1.26, 5.54) | 3.79 (1.55, 6.01) | 0.41 (0.14, 0.81) | 0.38 (0.1, 0.77) | 0.44 (0.15, 0.86) | 0.13 (0, 0.5) | 0.14 (0, 0.51) | 0.13 (0, 0.49) |
|  | 2000 | 4.62 (2.34, 6.93) | 4.39 (2.11, 6.7) | 4.83 (2.56, 7.15) | 4.05 (1.59, 6.64) | 3.83 (1.45, 6.46) | 4.26 (1.73, 6.8) | 0.42 (0.15, 0.86) | 0.4 (0.1, 0.83) | 0.44 (0.15, 0.89) | 0.15 (0, 0.57) | 0.16 (0, 0.59) | 0.14 (0, 0.55) |
|  | 2010 | 4.94 (2.67, 7.24) | 4.8 (2.54, 7.09) | 5.07 (2.79, 7.38) | 4.34 (1.86, 6.81) | 4.19 (1.73, 6.67) | 4.48 (1.98, 6.95) | 0.42 (0.15, 0.84) | 0.42 (0.1, 0.83) | 0.43 (0.15, 0.86) | 0.17 (0, 0.57) | 0.19 (0, 0.59) | 0.16 (0, 0.56) |
|  | 2017 | 5.12 (2.97, 7.31) | 5.06 (2.93, 7.25) | 5.18 (3.01, 7.38) | 4.53 (2.24, 6.79) | 4.46 (2.17, 6.7) | 4.6 (2.3, 6.87) | 0.41 (0.14, 0.79) | 0.41 (0.1, 0.79) | 0.41 (0.14, 0.8) | 0.18 (0, 0.54) | 0.19 (0, 0.55) | 0.17 (0, 0.53) |
| Sistan and Baluchistan | 1990 | 3.06 (1.26, 5.29) | 2.9 (1.15, 5.13) | 3.22 (1.36, 5.44) | 2.6 (0.86, 4.9) | 2.46 (0.78, 4.74) | 2.73 (0.94, 5.06) | 0.33 (0.11, 0.7) | 0.31 (0.1, 0.67) | 0.35 (0.12, 0.73) | 0.14 (0, 0.5) | 0.14 (0, 0.5) | 0.14 (0, 0.5) |
|  | 2000 | 3.66 (1.65, 5.97) | 3.56 (1.6, 5.86) | 3.75 (1.7, 6.07) | 3.18 (1.06, 6.13) | 3.09 (1.02, 6.09) | 3.27 (1.1, 6.17) | 0.37 (0.12, 0.81) | 0.36 (0.1, 0.81) | 0.37 (0.13, 0.81) | 0.11 (0, 0.59) | 0.11 (0, 0.59) | 0.11 (0, 0.58) |
|  | 2010 | 4.04 (1.9, 6.38) | 4.01 (1.9, 6.34) | 4.07 (1.89, 6.42) | 3.57 (1.34, 6.24) | 3.53 (1.34, 6.23) | 3.61 (1.34, 6.26) | 0.38 (0.13, 0.8) | 0.39 (0.1, 0.81) | 0.38 (0.13, 0.79) | 0.09 (0, 0.52) | 0.09 (0, 0.52) | 0.09 (0, 0.52) |
|  | 2017 | 4.24 (2.06, 6.49) | 4.25 (2.09, 6.52) | 4.24 (2.03, 6.47) | 3.8 (1.55, 6.15) | 3.79 (1.58, 6.18) | 3.8 (1.53, 6.13) | 0.37 (0.12, 0.75) | 0.38 (0.1, 0.77) | 0.36 (0.12, 0.73) | 0.08 (0, 0.46) | 0.08 (0, 0.46) | 0.08 (0, 0.46) |
| Tehran | 1990 | 4.56 (2.33, 6.81) | 4.42 (2.2, 6.67) | 4.69 (2.46, 6.95) | 3.4 (1.24, 5.71) | 3.34 (1.19, 5.64) | 3.46 (1.29, 5.78) | 0.42 (0.14, 0.84) | 0.39 (0.1, 0.8) | 0.44 (0.15, 0.87) | 0.74 (0.37, 1.11) | 0.69 (0.32, 1.06) | 0.79 (0.42, 1.16) |
|  | 2000 | 4.83 (2.54, 7.17) | 4.87 (2.56, 7.2) | 4.79 (2.52, 7.13) | 3.56 (1.23, 6.13) | 3.63 (1.27, 6.25) | 3.49 (1.2, 6.02) | 0.42 (0.14, 0.87) | 0.42 (0.1, 0.86) | 0.43 (0.14, 0.88) | 0.85 (0.43, 1.26) | 0.81 (0.39, 1.24) | 0.87 (0.46, 1.28) |
|  | 2010 | 4.88 (2.6, 7.22) | 5.07 (2.77, 7.41) | 4.69 (2.43, 7.05) | 3.6 (1.27, 6.06) | 3.78 (1.38, 6.32) | 3.42 (1.17, 5.82) | 0.41 (0.14, 0.84) | 0.43 (0.1, 0.86) | 0.4 (0.13, 0.82) | 0.86 (0.46, 1.26) | 0.86 (0.45, 1.27) | 0.87 (0.48, 1.26) |
|  | 2017 | 4.87 (2.7, 7.1) | 5.17 (3, 7.38) | 4.59 (2.43, 6.84) | 3.66 (1.4, 5.89) | 3.92 (1.53, 6.24) | 3.41 (1.27, 5.56) | 0.39 (0.13, 0.78) | 0.42 (0.1, 0.81) | 0.37 (0.12, 0.75) | 0.82 (0.46, 1.19) | 0.83 (0.46, 1.21) | 0.81 (0.45, 1.16) |
| Yazd | 1990 | 4.78 (2.55, 7.11) | 4.71 (2.46, 7.06) | 4.85 (2.63, 7.16) | 3.88 (1.58, 6.12) | 3.82 (1.53, 6.03) | 3.94 (1.62, 6.2) | 0.65 (0.31, 1.09) | 0.62 (0.3, 1.07) | 0.67 (0.33, 1.12) | 0.25 (0, 0.61) | 0.27 (0, 0.63) | 0.23 (0, 0.6) |
|  | 2000 | 5.31 (3, 7.68) | 5.27 (2.96, 7.66) | 5.34 (3.03, 7.71) | 4.35 (1.76, 6.89) | 4.33 (1.74, 6.87) | 4.38 (1.79, 6.91) | 0.64 (0.29, 1.14) | 0.61 (0.3, 1.12) | 0.67 (0.31, 1.17) | 0.31 (0, 0.73) | 0.33 (0, 0.75) | 0.3 (0, 0.71) |
|  | 2010 | 5.6 (3.3, 7.97) | 5.6 (3.3, 7.97) | 5.59 (3.3, 7.97) | 4.61 (2.08, 7.07) | 4.62 (2.08, 7.08) | 4.6 (2.08, 7.07) | 0.61 (0.28, 1.1) | 0.58 (0.3, 1.07) | 0.63 (0.29, 1.12) | 0.38 (0.02, 0.78) | 0.4 (0.03, 0.8) | 0.36 (0.01, 0.76) |
|  | 2017 | 5.73 (3.56, 7.99) | 5.77 (3.59, 8.02) | 5.7 (3.54, 7.95) | 4.76 (2.39, 7.03) | 4.79 (2.43, 7.05) | 4.72 (2.36, 7.01) | 0.57 (0.26, 1.02) | 0.55 (0.2, 1) | 0.59 (0.26, 1.04) | 0.41 (0.05, 0.78) | 0.43 (0.07, 0.8) | 0.39 (0.04, 0.76) |
| Zanjan | 1990 | 4.25 (2.12, 6.48) | 3.8 (1.76, 6.02) | 4.67 (2.47, 6.92) | 3.72 (1.63, 5.93) | 3.31 (1.34, 5.54) | 4.11 (1.9, 6.3) | 0.34 (0.12, 0.69) | 0.3 (0.1, 0.63) | 0.38 (0.14, 0.75) | 0.19 (0, 0.53) | 0.19 (0, 0.54) | 0.18 (0, 0.52) |
|  | 2000 | 4.76 (2.48, 7.07) | 4.4 (2.12, 6.71) | 5.09 (2.83, 7.41) | 4.21 (1.78, 6.74) | 3.89 (1.55, 6.44) | 4.52 (2.01, 7.02) | 0.36 (0.12, 0.75) | 0.33 (0.1, 0.7) | 0.4 (0.14, 0.8) | 0.18 (0, 0.58) | 0.18 (0, 0.59) | 0.18 (0, 0.58) |
|  | 2010 | 4.96 (2.66, 7.32) | 4.73 (2.43, 7.08) | 5.19 (2.89, 7.54) | 4.38 (1.91, 6.84) | 4.16 (1.72, 6.62) | 4.58 (2.08, 7.06) | 0.38 (0.12, 0.77) | 0.36 (0.1, 0.73) | 0.4 (0.13, 0.8) | 0.21 (0, 0.6) | 0.21 (0, 0.6) | 0.21 (0, 0.6) |
|  | 2017 | 5 (2.76, 7.26) | 4.87 (2.64, 7.14) | 5.13 (2.87, 7.37) | 4.39 (2.04, 6.69) | 4.27 (1.92, 6.56) | 4.5 (2.16, 6.82) | 0.38 (0.12, 0.75) | 0.37 (0.1, 0.73) | 0.39 (0.12, 0.77) | 0.23 (0, 0.6) | 0.23 (0, 0.6) | 0.23 (0, 0.6) |

*Data in parenthesis are 95% Uncertainty Interval.

**Appendix 5.**
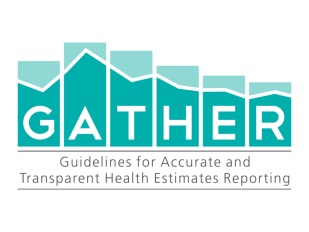
Checklist of information that should be included in new reports of global health estimates

| Item # | Checklist item | Reported on page # |
| --- | --- | --- |
| Objectives and funding | | |
| 1 | Define the indicator(s), populations (including age, sex, and geographic entities), and time period(s) for which estimates were made. | 4 |
| 2 | List the funding sources for the work. | None |
| Data Inputs | | |
| *For all data inputs from multiple sources that are synthesized as part of the study:* | | |
| 3 | Describe how the data were identified and how the data were accessed. | 5-6 |
| 4 | Specify the inclusion and exclusion criteria. Identify all ad-hoc exclusions. | 5 |
| 5 | Provide information on all included data sources and their main characteristics. For each data source used, report reference information or contact name/institution, population represented, data collection method, year(s) of data collection, sex and age range, diagnostic criteria or measurement method, and sample size, as relevant. | 5-7, Figure 1,  Supplementary materials |
| 6 | Identify and describe any categories of input data that have potentially important biases (e.g., based on characteristics listed in item 5). | None |
| *For data inputs that contribute to the analysis but were not synthesized as part of the study:* | | |
| 7 | Describe and give sources for any other data inputs. | None |
| *For all data inputs:* | | |
| 8 | Provide all data inputs in a file format from which data can be efficiently extracted (e.g., a spreadsheet rather than a PDF), including all relevant meta-data listed in item 5. For any data inputs that cannot be shared because of ethical or legal reasons, such as third-party ownership, provide a contact name or the name of the institution that retains the right to the data. | Supplementary materials |
| Data analysis | | |
| 9 | Provide a conceptual overview of the data analysis method. A diagram may be helpful. | 7-8 |
| 10 | Provide a detailed description of all steps of the analysis, including mathematical formulae. This description should cover, as relevant, data cleaning, data pre-processing, data adjustments and weighting of data sources, and mathematical or statistical model(s). | 7-8 |
| 11 | Describe how candidate models were evaluated and how the final model(s) were selected. | 7-8 |
| 12 | Provide the results of an evaluation of model performance, if done, as well as the results of any relevant sensitivity analysis. | 7-8 |
| 13 | Describe methods for calculating uncertainty of the estimates. State which sources of uncertainty were, and were not, accounted for in the uncertainty analysis. | 7-8 |
| 14 | State how analytic or statistical source code used to generate estimates can be accessed. | Upon request |
| Results and Discussion | | |
| 15 | Provide published estimates in a file format from which data can be efficiently extracted. | Supplementary Materials |
| 16 | Report a quantitative measure of the uncertainty of the estimates (e.g. uncertainty intervals). | 9-15 |
| 17 | Interpret results in light of existing evidence. If updating a previous set of estimates, describe the reasons for changes in estimates. | 18-20 |
| 18 | Discuss limitations of the estimates. Include a discussion of any modelling assumptions or data limitations that affect interpretation of the estimates. | 21 |
